# Efficient Photodynamic Killing of Gram-Positive Bacteria by Synthetic Curcuminoids

**DOI:** 10.3390/ijms21239024

**Published:** 2020-11-27

**Authors:** Sung-Jen Hung, Yi-An Hong, Kai-Yu Lin, Yi-Wen Hua, Chia-Jou Kuo, Anren Hu, Tzenge-Lien Shih, Hao-Ping Chen

**Affiliations:** 1 Institute of Medical Sciences, Tzu Chi University, Hualien 97004, Taiwan; md.hong@msa.hinet.net (S.-J.H.); amyhung840809@gmail.com (Y.-A.H.); 2 Department of Dermatology, Hualien Tzu Chi Hospital, Hualien 97002, Taiwan; 3 Department of Laboratory Medicine and Biotechnology, Tzu Chi University, Hualien 97004, Taiwan; 4 Department of Chemistry, Tamkang University, New Taipei City 25137, Taiwan; s930608eagle@gmail.com (K.-Y.L.); a0987719278@gmail.com (Y.-W.H.); b24631839@gmail.com (C.-J.K.); 5 Department of Biochemistry, Tzu Chi University, Hualien 97004, Taiwan; 6 Integration Center of Traditional Chinese and Modern Medicine, Hualien Tzu Chi Hospital, Hualien 97002, Taiwan

**Keywords:** bisdemethoxycurcumin, curcumin, curcuminoid, demethoxycurcumin, photodynamic inactivation, *Staphylococcus aureus*, *Staphylococcus epidermis*

## Abstract

In our previous study, we have demonstrated that curcumin can efficiently kill the anaerobic bacterium *Propionibacterium acnes* by irradiation with low-dose blue light. The curcuminoids present in natural plant turmeric mainly include curcumin, demethoxycurcumin, and bisdemethoxycurcumin. However, only curcumin is commercially available. Eighteen different curcumin analogs, including demethoxycurcumin and bisdemethoxycurcumin, were synthesized in this study. Their antibacterial activity against Gram-positive aerobic bacteria *Staphylococcus aureus* and *Staphylococcus epidermidis* was investigated using the photodynamic inactivation method. Among the three compounds in turmeric, curcumin activity is the weakest, and bisdemethoxycurcumin possesses the strongest activity. However, two synthetic compounds, (1*E*,6*E*)-1,7-bis(5-methylthiophen-2-yl)hepta-1,6-diene-3,5-dione and (1*E*,6*E*)-1,7-di(thiophen-2-yl)hepta-1,6-diene-3,5-dione, possess the best antibacterial activity among all compounds examined in this study. Their chemical stability is also better than that of bisdemethoxycurcumin, and thus has potential for future clinical applications.

## 1. Introduction

The emergence of drug-resistant bacteria has brought challenges to global public health and clinical treatments [1]. For all antibiotics currently used, a corresponding drug-resistant bacteria can be found [2]. The development of a new generation of antibiotics has become an increasingly important issue. However, progress in developing new antibiotics is dramatically slow [3]. More recently, antimicrobial photodynamic therapy (aPDT) appears to be a promising alternative approach and may become a new antimicrobial method [4]. Unlike traditional antibiotics, aPDT uses a photosensitizer or a nontoxic photoactivatable dye, visible light, and reactive oxygen to generate reactive oxygen species, like singlet oxygen or superoxide, to kill bacteria.

The antimicrobial activity of methylene blue, toluidine blue, rose bengal [5,6], indocyanine green [7], curcumin [8,9], and chlorin [10] induced by PDT has been reported previously. More recently, a synthetic compound, TTPy, has been proven to completely kill Gram-positive bacteria, namely *Staphylococcus aureus* and *Staphylococcus epidermidis*, under white light (60 mW/cm^2^) for 15 min [11]. However, as reported by our group previously, curcumin, a natural cooking spice isolated from *Curcuma longa* L rhizome, could kill the anaerobic Gram-positive bacteria *Propionibacterium acnes*, entirely under the irradiation of blue light (3 mW/cm^2^) for only 1 min [9]. Curcumin, therefore, appears to be an attractive aPDT agent. 

**Figure 1:**
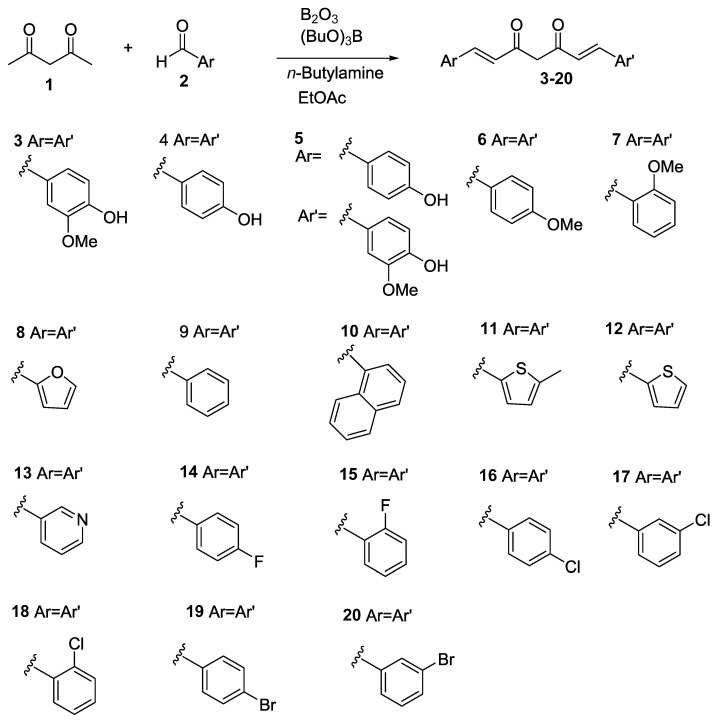
Chemical synthesis of curcuminoids **3**–**20**.

Curcuminoids in natural plant turmeric include curcumin (compound **3**), demethoxycurcumin (compound **4**), and bisdemethoxycurcumin (compound **5**) [12,13]. Curcumin is the primary form among them. At present, neither demethoxycurcumin nor bisdemethoxycurcumin is commercially available. Therefore, in contrast to curcumin, the biological activities of demethoxycurcumin and bisdemethoxycurcumin are relatively unknown. To further explore and improve the aPDT properties of curcumin, demethoxycurcumin, bisdemethoxycurcumin, and fifteen curcumin analogs (compounds **6**–**19** in Figure 1) were synthesized. The aPDT activities of the aforementioned compounds against Gram-positive bacteria *S. aureus* and *S. epidermidis* were investigated in this study.

## 2. Results and Discussion

### 2.1. Chemical Synthesis of Compounds **3**–**20**

Synthesis of symmetric curcuminoids **3**, **4**, and **6**–**20** followed Pabon’s method [14] (Figure 1). All of the starting materials were commercially available and inexpensive. One equivalent of 2,4-pentanedione was treated with two equivalents of corresponding aldehydes using B_2_O_3_ and (BuO)_3_B as complexing agents (see experimental). In contrast, the asymmetric curcuminoid **5** was applied to the strategy mentioned above, except one equivalent of aldehyde (Ar or Ar′) was added first. Notably, the subsequent aldehyde was added slowly via a syringe pump to afford a better yield of **5**. The NMR spectra of synthetic compounds are included in the Supplementary Figures S1–S36.

### 2.2. Antimicrobial Activity of Compound **3**–**20** with Blue Light Irradiation

**Figure 2:**
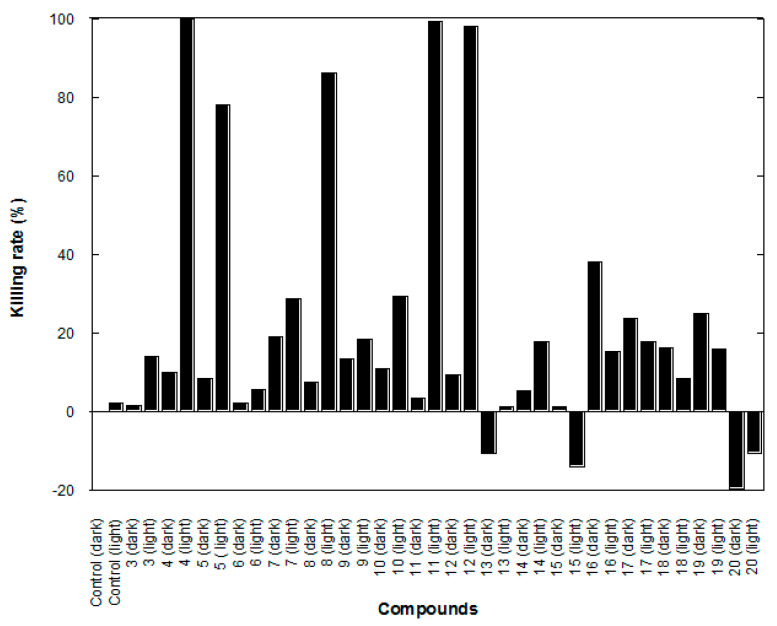
Bacterial killing activities of curcumin analogs on aerobic bacterium *Staphylococcus epidermidis*.

As shown in Figure 2, the antibacterial activities of compounds **3**–**20** against *S. epidermid* is were investigated. Compounds **4**, **5**, **8**, **11**, and **12** were the most effective among the eighteen compounds. The antibacterial activity of curcumin (compound **3**) was relatively weak, with a killing rate: 14.1%. In contrast to the previous report, the killing rate of curcumin against *P. acnes* is nearly 100% under similar experimental conditions. The possible reason for this difference is that *P. acnes* is an anaerobic bacterium, whereas *S. epidermidis* is aerobic. This result indicated that the antibacterial activity of demethoxycurcumin (compound **4**) and bisdemethoxycurcumin (compound **5**) was higher than that of curcumin (compound **3**), the primary isomer form in plant turmeric, under aerobic conditions.

Our previous results showed that curcumin’s photolytic products include vanillin, camphor, and acenaphthylene [9]. This result suggests that the formation of radicals is involved in this photolytic process. Generally, the antibacterial activity of compounds with halogen atom attached to the arene (compounds **14**–**20**) was low. Because the halogen atom is an electron-withdrawing group, this result implies that halogen’s attachment on those curcumin analogs is not conducive to these compounds’ photolysis. Previous studies have shown that curcumin binds effectively to the liposomal bilayer and locates preferentially in the hydrophobic acyl chain region [15]. Compounds **14**–**20** with halogen-substituted molecules should be much more hydrophobic than curcumin, altering the interactions with the bacterial lipid bilayer.

Different working concentrations of the compounds and bacterial strains were then used to compare the antibacterial activity of compounds **3**, **4**, **5**, **8**, **11**, and **12** (Table 1). Compounds **3**, **4**, and **5** are present in natural plant turmeric. It is interesting to note that synthetic compounds **8**, **11**, and **12** contain a hetero five-membered ring group. When the bacterial strain was switched to the other Gram-positive bacterium, *S. aureus*, the antibacterial activity of compounds **5** and **8** was significantly reduced. Furthermore, the concentration of the compounds **4**, **11**, and **12** was lowered to 0.5 ppm (Table 1). Thus, compounds **11** and **12** were the most effective among all the compounds tested in this study. The antibacterial activity of compound **11** on the Gram-negative bacterium *Escherichia coli* was also examined. The killing rate in the experimental and control groups was 18.1% and 17.1%, respectively, even when the working concentration of compound **11** was enhanced to 2 ppm. This result is in accordance with the previous report [11]. The synthetic compound TTPy can photodynamically kill Gram-positive bacteria *S. aureus* and *S. epidermidis*, but not the Gram-negative bacterium *E. coli*. All these results might come from the differences in cell envelop structures between Gram-positive and Gram-negative bacteria.

### 2.3. SEM Observation of Microbial Membrane Disruption after the Treatment of Compound **11** and Irradiation with Blue Light

**Figure 3:**
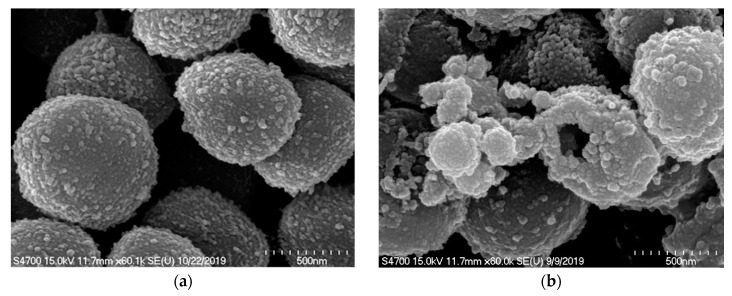

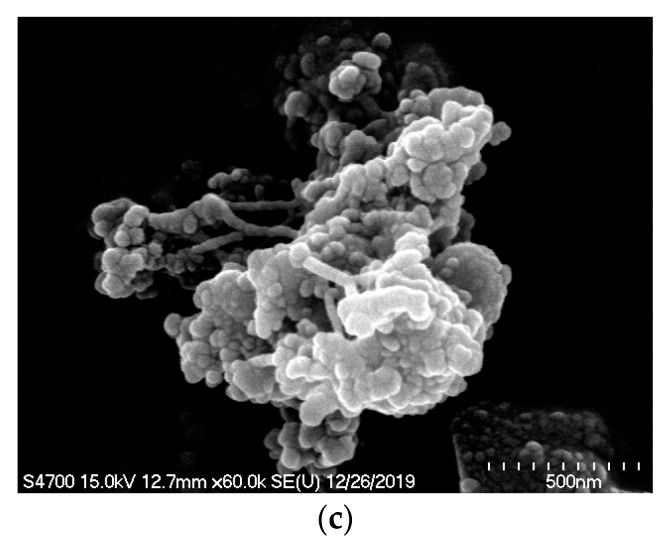
Scanning electron microscopy analysis of *Staphylococcus epidermidis* irradiated with blue light in the presence of 1 ppm compound **11**. (×60,000) The cell wall surface of *S. epidermidis* was severely damaged after the treatment. (**a**) Before irradiation with blue light, (**b**) Irradiation with blue light for 1 min, and (**c**) Irradiation with blue light for 5 min.

Our previous results showed that curcumin could disrupt *P. acnes* cell membranes after irradiation with blue light under anaerobic conditions by SEM [9]. Neither *S. aureus* nor *S. epidermidis* could be efficiently killed by curcumin under aerobic conditions in this study (Table 1), even though a previous report indicated that curcumin inhibited the growth of multi-resistant *S. aureus* by irradiation with LED for as long as 20 min [16]. SEM also examined the compound **11**-treated and blue light-irradiated *S. epidermidis* under aerobic conditions in this study. As shown in Figure 3, the bacterial cell membrane integrity was disrupted, and cellular morphology was altered. While the blue light irradiation time increases from 1 min to 5 min, the cell membrane damage also significantly increases.

### 2.4. Chemical Stability of Compounds **4**, **11**, and **12**

**Figure 4:**
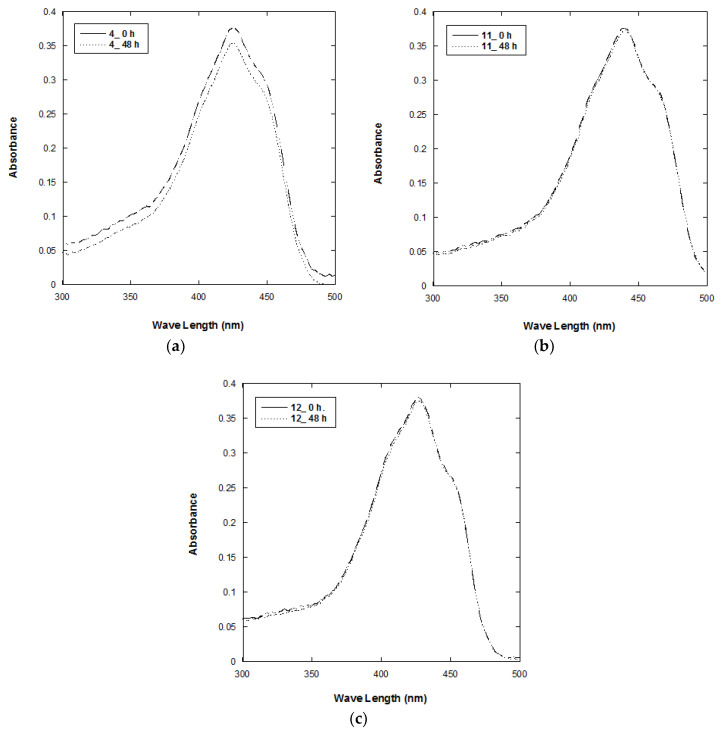
Absorption spectra of (**a**) compound **4** (20 ppm), (**b**) compound **11** (20 ppm), and (**c**) compound **12** (20 ppm), before and after storage in the dark for 48 h.

Curcumin easily undergoes autoxidation reactions in liquid at neutral-basic and alkaline pH [17]. The absorption spectra of compounds **4**, **11**, and **12** in the DMSO stock solution were recorded after storage at room temperature in the dark for 48 h. Their absorption spectra were recorded and shown in Figure 4. The maximum absorbance of compound **4** (λmax = 426 nm), **11** (λmax = 440 nm), and **12** (λmax = 426 nm) decreased 6.4%, 0.8%, and 1.3%, respectively. The color change of compounds **11** and **12** was not obvious. These results suggest that the chemical stability of compounds **11** and **12** is better than that of compound **4**. The NMR spectra of the degraded compound **4** were included in the Supplementary Figure S37.

## 3. Materials and Methods

### 3.1. Synthesis of the Curcumin Analogs **3**–**20**

All chemicals were purchased from Sigma-Aldrich (Shanghai, China) or Alfa-Aesar (Heysham, Lancashire, UK) companies and used without further purification. ^1^H and ^13^C NMR data were recorded on a Bruker 600 Ultrashield NMR spectrophotometer (Bruker, New Taipei City, Taiwan). The chemical shifts were reported in part per million (ppm) with the designated deuterium solvent relative to the residual solvent as internal standard (CDCl_3_, ^1^H: 7.26 ppm; ^13^C: 77.0 ppm.; CD_3_OD, ^1^H: 4.78 ppm; ^13^C: 49.15 ppm). Purification by flash column chromatography (SiliaFlash^®^ P60, 40–63 μm 60Å, SiliCycle^®^ Inc., Quebec City, QC, Canada) was performed on 230–400 mesh SiO_2_. The melting points were measured by a MP-2D apparatus (Fargo, New Taipei City, Taiwan) and not corrected. The mass data were obtained from JEOL JNS-700 (Akishima, Tokyo, Japan) by either EI or FAB and Bruker UltraFlex II for ESI (Bruker, New Taipei City, Taiwan).

#### 3.1.1. General Procedure in Preparation of Compounds **3**, **4**, and **6**–**20**

A mixture of acetylacetone (1.00 equiv.) and B_2_O_3_ (0.50 equiv.) in EtOAc (0.250 M) was heated at 50 °C for 30 min, followed by the addition of aldehyde (2.00 equiv.) and (BuO)_3_B (4.00 equiv.) in EtOAc (1.0 M), which was stirred at at 25 °C for 30 min before being added into the aforementioned solution. The resulting mixture was heated at 50 °C for 30 min, followed by the slow addition of *n*-butylamine (0.50 equiv.) in EtOAc (0.80 M), and then heated at 80 °C until reaction completion as indicated by TLC indication. Once the reaction was completed, HCl (1.0 N) was added and stirred for 30 min and then diluted with EtOAc and H_2_O. The organic layer was separated, dried by MgSO_4_, filtrated, and concentrated. 

#### 3.1.2. (1*E*,6*E*)-1,7-Bis(4-hydroxy-3-methoxyphenyl)hepta-1,6-diene-3,5-dione (**3**)

Vanillin (0.500 g, 3.29 mmol). Purification by flash column chromatography (EtOAc:Hexane = 1:3–1:1; EtOAc:Hexane = 1:2, *R_f_* = 0.4) afforded **3** (0.2179 g, 0.266 mmol) as a yellow solid. Yield: 36%. Mp 182–186 °C ^1^H NMR (600 MHz, CD_3_OD): *δ*7.56(d, *J* = 15.7 Hz, 2H), 7.20 (s, 2H), 7.09 (d, *J* = 7.9 Hz, 2H), 6.80 (d, *J* = 8.1 Hz, 2H), 6.61 (d, *J* = 15.7 Hz, 2H), 3.90 (s, 6H). ^13^C NMR (150 MHz, CD_3_OD): *δ*185.0, 184.8, 161.2, 150.5, 149.5, 142.2, 142.0, 131.3, 128.7, 128.1, 124.2, 122.3, 122.1, 117.0, 116.7, 111.9, 56.6. HRMS (FAB) calculated for C_21_H_20_O_6_ ([M]^+^): 368.1260. Found: 368.1261. 

#### 3.1.3. (1*E*,6*E*)-1,7-Bis(4-hydroxyphenyl)hepta-1,6-diene-3,5-dione (**4**)

4-Hydroxybenzaldehyde (0.500 g, 4.10 mmol). Purification by flash column chromatography (EtOAc:Hexane = 1:3–1:1; EtOAc:Hexane = 1:3, *R_f_* = 0.3) afforded **4** (0.218 g, 0.592 mmol) as a red solid. Yield: 42%. Mp 232–236 °C. ^1^H NMR (600 MHz, CD_3_OD): *δ*7.55 (d, *J* = 15.8 Hz, 2H), 7.50 (dd, *J* =7.8, 4H), 6.81 (d, *J* = 7.8 Hz, 4H), 6.57 (d, *J* = 15.8 Hz, 4H). ^13^C NMR (150 MHz, CD_3_OD): *δ*184.8, 161.1, 141.9, 131.2, 128.0, 122.0, 117.0. HRMS (FAB) calculated for C_19_H_16_O_4_ ([M]^+^): 308.1049. Found: 308.1049.

#### 3.1.4. (1*E*,6*E*)-1-(4-hydroxy-3-methoxyphenyl)-7-(4-hydroxyphenyl)Hepta-1,6-diene-3,5-dione (**5**)

Followed the general procedure except the vanillin was used half equivalent relative to acetyl acetone. The resulting mixture was purified by flash column chromatography (EtOAc:Hexane= 1:2–1:1) to afford an intermediate as a yellow solid in 28% yield. This yellow solid (0.200 g, 0.850 mmol) was applied the general procedure and used the equivalent amount of 4-hydroxybenzaldehyde (0.207 g, 1.7 mmol). At the end of reaction time, purification by flash column chromatography (EtOAc:Hexane = 1:3–1:1; EtOAc:Hexane = 1:2, *R_f_* = 0.3) afforded **5** (0.090 g, 0.266 mmol) as a red solid. Yield: 31%. Mp 172–174 °C. ^1^H NMR (600 MHz, CD_3_OD): *δ*7.53 (d, *J* = 15.8 Hz, 2H), 7.52 (d, *J* = 15.8 Hz, 2H), 7.44 (d, *J* = 8.4 Hz, 1H), 7.15 (s, 1H), 7.06 (d, *J* = 8.2 Hz, 1H), 6.80–6.78 (m, 3H), 6.57 (d, *J* = 15.8 Hz, 1H),6.54 (d, J = 15.8 Hz, 1H), 4.86 (s, 2H), 3.87 (s, 3H). ^13^C NMR (150 MHz, CD_3_OD): *δ*185.0, 184.8, 161.2, 150.5, 149.5, 142.2, 142.0, 131.3, 128.7, 124.2, 122.4, 122.1, 117.0, 116.7, 111.9, 56.6. HRMS (ESI) calculated for C_20_H_19_O_5_ ([M+H]^+^): 339.1232. Found: 339.1227.

#### 3.1.5. (1*E*,6*E*)-1,7-Bis(4-methoxyphenyl)hepta-1,6-diene-3,5-dione (**6**)

*p*-Anisaldehyde (0.447 g, 3.282 mmol). Purification by flash column chromatography (EtOAc:Hexane = 1:5–1:1; EtOAc:Hexane = 1:3, *R_f_* = 0.3) afforded **6** (0.379 g, 1.128 mmol) as a red solid. Yield: 69%. Mp 163–165 °C. ^1^H NMR (600 MHz, CDCl_3_): *δ*7.62 (d, *J* = 15.8 Hz, 2H), 7.51 (d, *J* = 8.8 Hz, 2H), 6.91 (d, *J* = 8.8 Hz, 2H), 6.50 (d, *J* = 15.8 Hz, 2H), 5.79 (s, 1H), (s, 1H), 3.84 (s, 6H). ^13^C NMR (150 MHz, CDCl_3_): *δ*183.3, 161.3, 140.1, 129.7, 127.8, 121.8, 114.4, 101.3, 55.4. HRMS (ESI) calculated for C_21_H_21_O_4_ ([M+H]^+^): 337.1440. Found: 337.1434. 

#### 3.1.6. (1*E*,6*E*)-1,7-Bis(2-methoxyphenyl)hepta-1,6-diene-3,5-dione (**7**)

2-Methoxybenzaldehyde (2.723 g, 20.000 mmol). Purification by flash column chromatography (EtOAc:Hexane = 1:3–1:1; EtOAc:Hexane = 1:2, *R_f_* = 0.3) afforded **7** (1.033 g, 3.071 mmol) as a yellow solid. Yield: 31%. Mp 114–116 °C. ^1^H NMR (600 MHz, CDCl_3_): *δ*7.99 (d, *J* = 16.0 Hz, 2H), 7.55 (d, *J* = 7.6 Hz, 2H), 7.34 (t, *J* = 7.8 Hz, 2H), 6.97 (t, *J* = 7.5 Hz, 2H), 6.92 (d, *J* = 8.2 Hz, 2H), 6.72 (d, *J* = 16.0 Hz, 2H), 5.88 (s, 1H), 3.90 (s, 6H). ^13^C NMR (150 MHz, CDCl_3_): *δ*183.8, 158.4, 135.7, 131.2, 128.6, 124.8, 124.1, 120.7, 111.2, 101.5, 55.5. HRMS (FAB) calculated for C_21_H_20_O_4_ ([M]^+^): 336.1362. Found: 336.1359.

#### 3.1.7. (1*E*,6*E*)-1,7-Di(furan-2-yl)hepta-1,6-diene-3,5-dione (**8**)

2-Furaldehyde (0.510 g, 5.308 mmol). Purification by flash column chromatography (EtOAc:Hexane = 1:10–1:5; EtOAc:Hexane = 1:10, *R_f_* = 0.4) afforded **8** (0.0691 g, 0.270 mmol) as an orange-yellow solid. Yield: 10%. Mp 128–129 °C. ^1^H NMR (600 MHz, CDCl_3_): *δ*7.48 (s, 2H), 7.40 (d, *J* = 15.5 Hz, 2H), 6.60 (d, *J* = 3.4 Hz, 2H), 6.51 (d, *J* = 15.5 Hz, 2H), 6.40 (dd, *J* = 2.9, 1.4 Hz, 2H), 5.74 (s, 1H). ^13^C NMR (150 MHz, CDCl_3_): *δ*182.7, 151.7, 144.7, 126.8, 121.8, 114.8, 112.5, 102.3. HRMS (FAB) calculated for C_15_H_12_O_4_ ([M]^+^): 256.0736. Found: 256.0736.

#### 3.1.8. (1*E*,6*E*)-1,7-Diphenylhepta-1,6-diene-3,5-dione (**9**)

Benzaldehyde (2.122 g, 20.000 mmol). Purification by flash column chromatography (EtOAc:Hexane = 1:3–1:1; EtOAc:Hexane = 1:2, *R_f_* = 0.8) afforded **9** (2.016 g, 7.300 mmol) as a yellow solid. Yield: 73%. Mp 154–155 °C. ^1^H NMR (600 MHz, CDCl_3_): *δ*7.67 (d, *J* = 15.9 Hz, 2H), 7.56 (d, *J* = 6.6 Hz, 4H), 7.42–7.36 (m, 6H). 6.54 (d, *J* = 15.8 Hz, 2H), 5.86 (s, 1H). ^13^C NMR (150 MHz, CDCl_3_): *δ*183.3, 140.6, 135.0, 130.1, 128.9, 128.1, 124.1, 101.8. HRMS (FAB) calculated for C_19_H_16_O_2_ ([M]^+^): 276.1150. Found: 276.1150.

#### 3.1.9. (1*E*,6*E*)-1,7-Di(naphthalen-1-yl)hepta-1,6-diene-3,5-dione (**10**)

1-Naphthaldehyde (0.500 g, 3.201 mmol). Purification by flash column chromatography (EtOAc:Hexane = 1:10–1:5; EtOAc:Hexane = 1:10, *R_f_* = 0.5) afforded **10** (0.156 g, 0.4115 mmol) as a yellow solid. Yield: 26%. Mp 177–180 °C. ^1^H NMR (600 MHz, CDCl_3_): *δ*8.55 (d, *J* = 15.6 Hz, 2H), 8.27 (d, *J* = 8.4 Hz, 2H), 7.90 (t, *J* = 8.2 Hz, 4H), 7.82 (d, *J* = 7.2 Hz, 2H), 7.60 (td, *J* = 8.2, 1.1 Hz, 2H), 7.55 (td, *J* = 8.0, 1.0 Hz, 2H), 7.51 (t, *J* = 7.6 Hz, 2H), 6.76 (d, *J* = 15.6 Hz, 2H), 5.95 (s, 1H). ^13^C NMR (150 MHz, CDCl_3_):*δ*183.3, 137.5, 133.8, 132.4, 131.6, 130.5, 128.7, 126.9, 126.6, 126.3, 125.5, 124.9, 123.5, 102.2. HRMS (FAB) calculated for C_27_H_20_O_2_ ([M]^+^): 376.1463. Found: 376.1466.

#### 3.1.10. (1*E*,6*E*)-1,7-Bis(5-methylthiophen-2-yl)hepta-1,6-diene-3,5-dione (**11**)

5-Methylthiophene-2-carboxaldehyde (2.524 g, 20.003 mmol). Purification by flash column chromatography (EtOAc:Hexane = 1:4–1:1; EtOAc:Hexane = 1:3, *R_f_* = 0.6) afforded **11** (1.138 g, 3.601 mmol) as a brown solid. Yield: 36%. Mp 140–141 °C. ^1^H NMR (600 MHz, CDCl_3_): *δ*7.67 (d, *J* = 15.3 Hz, 2H), 7.05 (d, *J* = 3.3 Hz, 2H), 6.71 (d, *J* = 3.2 Hz, 2H), 6.26 (d, *J* = 15.4 Hz, 2H), 5.68 (s, 1H), 2.50 (s, 6H). ^13^C NMR (150 MHz, CDCl_3_): *δ*182.7, 144.0, 138.6, 133.3, 131.5, 126.7, 121.7, 101.4, 15.8. HRMS (FAB) calculated for C_17_H_16_O_2_S_2_ ([M]^+^): 316.0592. Found: 316.0593.

#### 3.1.11. (1*E*,6*E*)-1,7-Di(thiophen-2-yl)hepta-1,6-diene-3,5-dione (**12**)

2-Thiophenecarboxaldehyde (1.116 g, 9.951 mmol). Purification by flash column chromatography (CH_2_Cl_2_:Hexane = 3:1–20:1; CH_2_Cl_2_:Hexane = 1:1, *R_f_* = 0.5) afforded **12** (0.317 g, 1.101 mmol) as a brown solid. Yield: 22%. Mp 195–197 °C. ^1^H NMR (600 MHz, CDCl_3_): *δ*7.75 (d, *J* = 15.4 Hz, 2H), 7.38 (d, *J* = 5.0 Hz, 2H), 7.26 (d, *J* = 4.2 Hz, 2H), 7.06 (dd, *J* = 5.0, 3.6 Hz, 2H), 6.41 (d, *J* = 15.4 Hz, 2H), 5.74 (s, 1H). ^13^C NMR (150 MHz, CDCl_3_): *δ*182.7, 140.5, 133.1, 130.9, 128.4, 123.0, 101.8. HRMS (FAB) calculated for C_15_H_12_O_2_S_2_ ([M]^+^): 288.0279. Found: 288.0279.

#### 3.1.12. (1*E*,6*E*)-1,7-Di(pyridin-3-yl)hepta-1,6-diene-3,5-dione (**13**)

3-Pyridinecarboxaldehyde (1.000 g, 9.340 mmol). Purification by flash column chromatography (EtOAc:Hexane = 1:5–1:1; EtOAc:Hexane = 1:5, *R_f_* = 0.5) afforded **13** (0.602 g, 2.35 mmol) as a brown solid. Yield: 47%. Mp 180–181 °C. ^1^H NMR (600 MHz, CDCl_3_): *δ*8.79 (s, 2H), 8.60 (d, *J* = 4.1 Hz, 2H), 7.86 (d, *J* = 7.8 Hz, 2H), 7.66 (d, *J* = 15.9 Hz, 2H), 7.34 (dd, *J* = 7.7, 4.9 Hz, 2H), 6.70 (d, *J* = 15.9 Hz, 2H), 5.89 (s, 1H). ^13^C NMR (150 MHz, CDCl_3_): *δ*182.8, 150.8, 149.7, 137.2, 134.3, 130.7, 125.8, 123.8, 102.2. HERMS (FAB) calculated for C_15_H_12_O_4_ ([M]^+^): 256.0736. Found: 256.0736.

#### 3.1.13. (1*E*,6*E*)-1,7-Bis(4-fluorophenyl)hepta-1,6-diene-3,5-dione (**14**)

4-Fluorobenzaldehyde (1.240 g, 9.991 mmol). Purification by flash column chromatography (EtOAc:Hexane = 1:3–1:1; EtOAc:Hexane = 1:3, *R_f_* = 0.7) afforded **14** (0.312 g, 0.998 mmol) as a pale-yellow solid. Yield: 20%. Mp 172–173 °C. ^1^H NMR (600 MHz, CDCl_3_): *δ*7.63 (d, *J* = 15.8 Hz, 2H), 7.55 (d, *J* = 5.5 Hz, 2H), 7.54 (d, *J* = 5.5 Hz, 2H), 7.09 (d, *J* = 8.5 Hz, 2H), 7.06 (d, *J* = 11.5 Hz, 2H), 6.54 (d, *J* = 15.7 Hz, 2H), 5.81 (s,1H). ^13^C NMR (150 MHz, CDCl_3_): *δ*183.1, 163.8 (^1^*J*_C-F_ = 249.7 Hz), 139.4, 131.2. 129.9 (^3^*J*_C-F_ = 8.3 Hz), 123.7, 116.1 (^2^*J*_C-F_ = 21.8 Hz), 101.8. HERMS (FAB) calculated for C_19_H_14_F_2_O_2_ ([M]^+^): 312.0962. Found: 312.0963.

#### 3.1.14. (1*E*,6*E*)-1,7-Bis(2-fluorophenyl)hepta-1,6-diene-3,5-dione (**15**)

2-Fluorobenzaldehyde (0.408 g, 3.290 mmol). Purification by flash column chromatography (EtOAc:Hexane = 1:10–1:1; EtOAc:Hexane = 1:2, *R_f_* = 0.6) afforded **15** (0.323 g, 1.04 mmol) as a yellow solid. Yield: 63%. Mp 100–102 °C. ^1^ H NMR (600 MHz, CDCl_3_): *δ*7.78 (d, *J* = 16.1 Hz, 2H), 7.57 (td, *J* = 7.6, 1.6 Hz, 2H), 7.36–7.33 (m, 2H), 7.18 (t, *J* = 7.7 Hz, 2H), 7.11 (dd, *J* = 9.0, 8.3 Hz, 2H), 6.76 (d, *J* = 16.1 Hz, 2H), 5.90 (s, 1H). ^13^C NMR (150 MHz, CDCl_3_): *δ*183.3, 161.5 (^1^*J*_C-F_ = 252.0 Hz), 133.4, 131.4 (^3^*J*_C-F_ = 7.5 Hz), 129.2, 126.6 (^3^*J*_C-F_ = 6.0 Hz), 124.4 (^4^*J*_C-F_ = 3.0 Hz), 116.2 (^2^*J*_C-F_ = 11.0 Hz), 116.2 (^2^*J*_C-F_ = 21.0 Hz). HRMS (ESI) calculated for C_19_H_15_F_2_O_2_ ([M+H]^+^): 313.1040. Found: 313.1038.

#### 3.1.15. (1*E*,6*E*)-1,7-Bis(4-chlorophenyl)hepta-1,6-diene-3,5-dione (**16**)

4-Chlorobenzaldehyde (2.811 g, 20.000 mmol). Purification by flash column chromatography (EtOAc:Hexane = 1:4–1:1; EtOAc:Hexane = 1:4, *R_f_* = 0.6) afforded **16** (1.800 g, 5.21 mmol) as a yellow solid. Yield: 52%. Mp 165–166 °C. ^1^H NMR (600 MHz, CDCl_3_): *δ*7.61 (d, *J* = 15.8 Hz, 2H), 7.49 (d, *J* = 8.4 Hz, 4H), 7.37 (d, *J* = 8.4 Hz, 4H), 6.59 (d, *J* = 15.8 Hz, 2H), 5.83 (s, 1H). ^13^C NMR (150 MHz, CDCl_3_): *δ*183.0, 129.3, 136.0, 133.4, 129.3, 129.2, 124.5, 102.2. HRMS (FAB) calculated for C_19_H_14_Cl_2_O_2_ 344.0317. Found: 344.0317.

#### 3.1.16. (1*E*,6*E*)-1,7-Bis(3-chlorophenyl)hepta-1,6-diene-3,5-dione (**17**)

3-Chlorobenzaldehyde (2.811 g, 20.000 mmol). Purification by flash column chromatography (EtOAc:Hexane = 1:5–1:1; EtOAc:Hexane = 1:1, *R_f_* = 0.6) afforded **17** (1.417 g, 4.107 mmol) as an amorphous yellow solid. Yield: 41%. Mp 153–154 °C. ^1^H NMR (600 MHz, CDCl_3_): *δ*7.57 (d, *J* = 15.8 Hz, 2H), 7.52 (s, 2H), 7.39 (d, *J* = 7.0 Hz, 2H), 7.35–7.25 (m, 4H), 6.60 (d, *J* = 15.8 Hz, 2H), 5.83 (s, 1H). ^13^C NMR (150 MHz, CDCl_3_): *δ*182.9, 139.1, 136.7, 134.9, 130.1, 129.9, 127.6, 126.4, 125.2, 102.3. HRMS (ESI) calculated for C_19_H_15_Cl_2_O_2_ ([M+H]^+^): 345.0449. Found: 345.0448.

#### 3.1.17. (1*E*,6*E*)-1,7-Bis(2-chlorophenyl)hepta-1,6-diene-3,5-dione (**18**)

2-Chlorobenzaldehyde (3.390 g, 24.116 mmol). Purification by flash column chromatography (EtOAc:Hexane = 1:4–1:1; EtOAc:Hexane = 1:4, *R_f_* = 0.6) afforded **18** (0.757 g, 2.194 mmol) as a yellow solid. Yield: 18%. Mp 147–148 °C. ^1^H NMR (600 MHz, CDCl_3_): *δ*8.06 (d, *J* = 15.8 Hz), 7.67–7.64 (m, 2H), 7.43–7.41 (m, 2H), 7.32–7.27 (m*,* 4H), 6.62 (d, *J* = 15.8 Hz, 2H), 5.91 (s, 1H). ^13^C NMR (150 MHz, CDCl_3_): *δ*183.1, 136.5, 135.1, 133.1, 130.8, 130.3, 127.5, 127.0, 126.5, 101.7. HRMS (ESI) calculated for C_19_H_15_Cl_2_O_2_ ([M+H]^+^): 345.0449. Found: 345.0444.

#### 3.1.18. (1*E*,6*E*)-1,7-Bis(4-bromophenyl)hepta-1,6-diene-3,5-dione (**19**)

4-Bromobenzaldehyde (0.609 g, 3.292 mmol). Purification by flash column chromatography (EtOAc:Hexane = 1:10–1:1; EtOAc:Hexane = 1:2, *R_f_* = 0.6) afforded **19** (0.429 g, 0.993 mmol) as a yellow solid. Yield: 60%. Mp 233–235 °C. ^1^H NMR (600 MHz, CDCl_3_): *δ*7.60 (d, *J* *=* 15.8 Hz, 2H), 7.53 (d, *J* = 8.4 Hz, 4H), 7.42 (d, *J* = 8.4 Hz, 4H), 6.61 (d, *J* = 15.8 Hz, 2H), 5.83 (s, 1H). ^13^C NMR (150 MHz, CDCl_3_): *δ*183.0, 139.4, 133.9, 132.2, 129.5, 124.6, 124.4, 102.1. HRMS (ESI) calculated for C_19_H_15_Br_2_O_2_ ([M+H]^+^): 432.9439. Found: 432.9434.

#### 3.1.19. (1*E*,6*E*)-1,7-Bis(3-bromophenyl)hepta-1,6-diene-3,5-dione (**20**)

3-Bromobenzaldehyde (0.500 g, 2.702 mmol). Purification by flash column chromatography (EtOAc:Hexane = 1:10–1:3; EtOAc:Hexane = 1:10, *R_f_* = 0.4) afforded **20** (0.429 g, 0.993 mmol) as a yellow solid. Yield: 74%. Mp 152–154 °C. ^1^H NMR (600 MHz, CDCl_3_): *δ*7.71 (t, *J* = 1.7 Hz, 2H), 7.58 (d, *J* = 15.8 Hz, 2H), 7.51–7.49 (m, 2H), 7.47 (d, *J* = 7.9 Hz, 2H), 7.28 (d, *J* = 7.9 Hz, 2H), 6.21 (d, *J* = 16.2 Hz, 2H), 5.84 (s, 1H). ^13^C NMR (150 MHz, CDCl_3_): *δ*182.9, 139.1, 137.1, 132.9, 130.6, 130.4, 126.9, 125.3, 123.1, 103.3. HRMS (ESI) calculated for C_19_H_15_Br_2_O_2_ ([M+H]^+^): 432.9434. Found: 432.9437.

### 3.2. Photodynamic Antibacterial Studies

The photo-irradiation system for the microbial viability experiments was reported previously [9]. The blue light intensity was 3.0 mW/cm^2^ using a DC 5V power supply. The LED (Vetalux Company, Tainan, Taiwan) emission spectra were from 410 to 510 nm with λmax = 462 nm. *S. epidermidis* TCU-1 BCRC 81267 and *S. aureus* subsp. aureus TCU-2 BCRC 81268 were obtained from the Bioresource Collection and Research Center, Hsinchu, Taiwan. *Escherichia coli* was provided by Professor Kai-Chih Chang (Department of Laboratory Medicine and Biotechnology, Tzu Chi University, Taiwan). All bacterial strains were cultured in LB medium (BD Biosciences, San Jose, CA, USA) at 37 °C until OD_600_ reached 1.0. The number of bacteria was about 10^9^ CFU/mL.

Curcumin and its analogs were dissolved in 100% DMSO (Sigma-Aldrich, Shanghai, China), and the concentration of this stock was 2000 ppm. These DMSO stocks were diluted with LB medium. A total of 2 mL bacterial cultures was treated with 0.5 or 1 ppm of curcumin and its synthetic derivatives, and irradiated with 3.0 mW/cm^2^ of blue light for 1 min (equivalent to radiant exposure of 0.18 J/cm^2^). The cultures were then serially diluted before streaking and spreading on LB agar plates. After incubation at 37 °C overnight, the microbial colonies were counted, and the killing ratio was calculated as follows:

(1) $$\;\mathrm{Killing}\;\mathrm{ratio}\;\left(\%\right)\;=\left\{1-\;\frac{T_{\left(\mathrm{CFU}/\mathrm{mL}\right)}}{C_{\left(\mathrm{CFU}/\mathrm{mL}\right)}}\right\}\times 100\%$$

where T is the colony number of the curcumin and its synthetic derivatives-treated group, and C is the colony number of the control group (DMSO only) without light irradiation.

### 3.3. Scanning Electron Microscope (SEM) Observation of Microbial Membrane Disruption

After the treatment of compound **11** and blue light irradiation, the surface morphological changes in *S. epidermidis* cells were examined using Hitachi S-4700 SEM (Hitachi, Tokyo, Japan). The preparation for SEM samples was described previously [9]. A total of 2 mL 10^9^ CFU/mL bacterial culture was treated with 1 ppm compound **11** and irradiated with blue light for 1 or 5 min.

### 3.4. Chemical Stability of Compounds **4**, **11**, and **12**

The 20 ppm DMSO solutions of compounds **4**, **11**, and **12** were prepared and stored in the dark at room temperature for 48 h. Before and after storage, the UV-visible spectra of compounds **1**, **8**, and **9** were recorded in the wavelength range of 220–750 nm.

### 3.5. Statistical Analysis

The experiments were performed in triplicate, and the data are expressed as mean ± standard deviation of three individual experiments. The data were assessed by analysis of variance (ANOVA) using SPSS Statistics (IBM, Armonk, NY, USA). *p* < 0.05 was considered significant.

## 4. Conclusions

The antibacterial activity of eighteen curcumin analogs against Gram-positive aerobic bacteria *S. aureus* and *S. epidermidis* was investigated by the photodynamic inactivation method. The antibacterial activity of all analogs containing halogen atom (compounds **14** to **20**) was low. The reason for this is still not clear. Two compounds, (1*E*,6*E*)-1,7-bis(5-methylthiophen-2-yl)hepta-1,6-diene-3,5-dione (compound **11**) and (1*E*,6*E*)-1,7-di(thiophen-2-yl)hepta-1,6-diene-3,5-dione (compound **12**), had the strongest antibacterial activity. Their chemical stability was also better than that of natural curcuminoids. Because natural curcuminoids are easily oxidized in solution, this feature makes these two compounds potentially useful for future clinical applications.

## Figures and Tables

**Table 1:** The killing efficiency of compounds **3**, **4**, **5**, **8**, **11**, and **12** against *Staphylococcus aureus* and *S. epidermidis* with 1 min blue light irradiation.

Bacterial Strain	*S. aureus*	*S. epidermidis*	*S. epidermidis*
Working Concentration	1 ppm	1 ppm	0.5 ppm
Control (in dark)	N/A	N/A	N/A
Control (with BL irradiation)	2.9 ± 2.2	7.7 ± 10.0	7.6 ± 4.8
**3** (in dark)	12.0 ± 12.0	2.6 ± 12.4	
**3** (with BL irradiation)	18.6 ± 6.3	14.9 ± 1.3	
**4** (in dark)	19.3 ± 13.6	−5.0 ± 19.6	5.9 ± 7.0
**4** (with BL irradiation)	100 ± 0	98.5 ± 1.5	22.3 ± 3.2
**5** (in dark)	17.1 ± 2.0	12.5 ± 4.4	
**5** (with BL irradiation)	31.0 ± 2.5	71.1+ 9.8	
**8** (in dark)	0.7 ± 6.2	4.3 ± 8.8	
**8** (with BL irradiation)	27.1 ± 18.0	91.8 ± 7.3	
**11** (in dark)	−2.0 ± 5.2	6.7 ± 4.1	26.0 ± 11.3
**11** (with BL irradiation)	99.7 ± 0.3	99.8 ± 0.2	97.3 ± 0.7
**12** (in dark)	13.5 ± 3.9	4.2 ± 4.7	7.9 ± 9.0
**12** (with BL irradiation)	100 ± 0	99.7 ± 0.3	87.8 ± 12.2

All experiments were performed in triplicate. All data are expressed as the mean ± standard deviation. BL: blue light.

## Supplementary Materials

The following are available online at https://www.mdpi.com/1422-0067/21/23/9024/s1.

## Abbreviations

aPDT	antimicrobial photodynamic therapy
BL	Blue light
SEM	Scanning Electron Microscope
