# Supplementary material for: Efficient Photodynamic Killing of Gram-Positive Bacteria by Synthetic Curcuminoids
Source: Int J Mol Sci. 2020 Nov 27;21(23):9024. doi: 10.3390/ijms21239024 (PMC7730963; doi:10.3390/ijms21239024)
Supplement: Supplementary file 1 [file ijms-21-09024-s001.pdf]

Supplementary Materials

Efficient Photodynamic Killing of Gram-Positive Bacteria by Synthetic Curcuminoids

Sung-Jen Hung [1,2], Yi-An Hong [1,3], Kai-Yu Lin [4], Yi-Wen Hua [4], Chia-Jou Kuo [4], Anren Hu [1,3,*], Tzenge-Lien Shih [4,*] and Hao-Ping Chen [1,5,6,*]

1. Institute of Medical Sciences, Tzu Chi University, Hualien 97004, Taiwan; md.hong@msa.hinet.net (S.-J.H.); amyhung840809@gmail.com (Y.-A.H.)
2. Department of Dermatology, Hualien Tzu Chi Hospital, Hualien 97002, Taiwan
3. Department of Laboratory Medicine and Biotechnology, Tzu Chi University, Hualien 97004, Taiwan
4. Department of Chemistry, Tamkang University, New Taipei City 25137, Taiwan; s930608eagle@gmail.com (K.-Y.L.); a0987719278@gmail.com (Y.-W.H.); b24631839@gmail.com (C.-J.K.)
5. Department of Biochemistry, Tzu Chi University, Hualien 97004, Taiwan
6. Integration Center of Traditional Chinese and Modern Medicine, Hualien Tzu Chi Hospital, Hualien 97002, Taiwan

* Correspondence: anren@gms.tcu.edu.tw (A.H.); tlshih@mail.tku.edu.tw (T.-L.S.); hpchen@mail.tcu.edu.tw (H.-P.C.); Tel.: +886-3-856-5301 ext. 2335 (A.H); +886-2-8631-5024 (T.-L.S.); +886-3-856-5301 ext. 2433 (H.-P.C.)

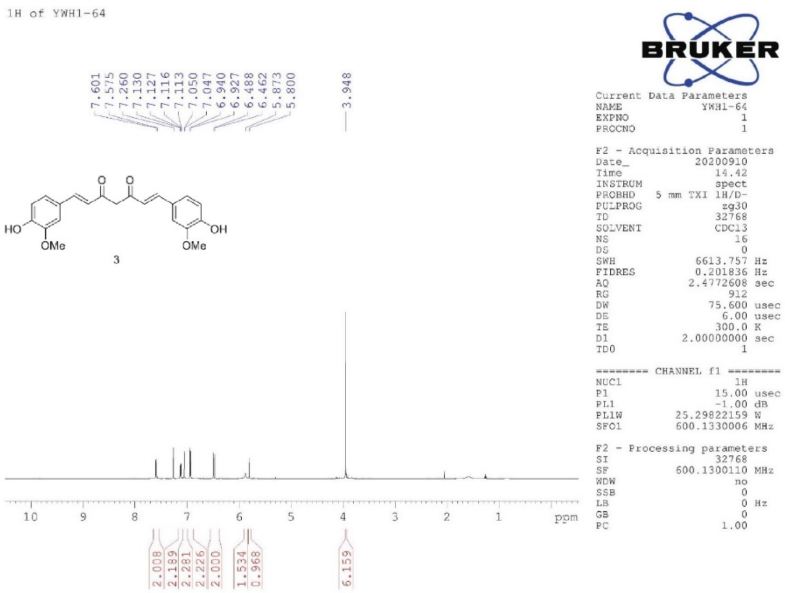

Figure S1. ^{1}H NMR (600 MHz, $CDCl_3$) of compound **3**.

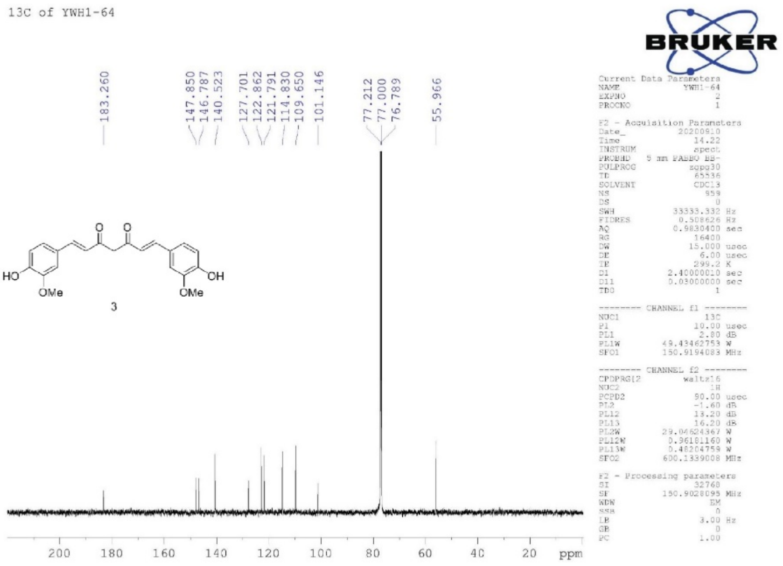

Figure S2. ^{13}C NMR (150 MHz, $CDCl_3$) of compound **3**.

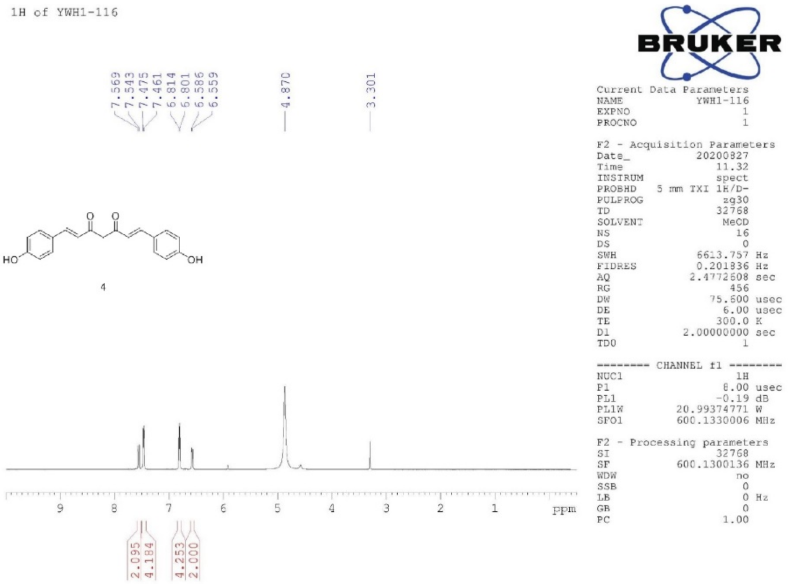

Figure S3. ^{1}H NMR (600 MHz, CD_3OD) of compound **4**.

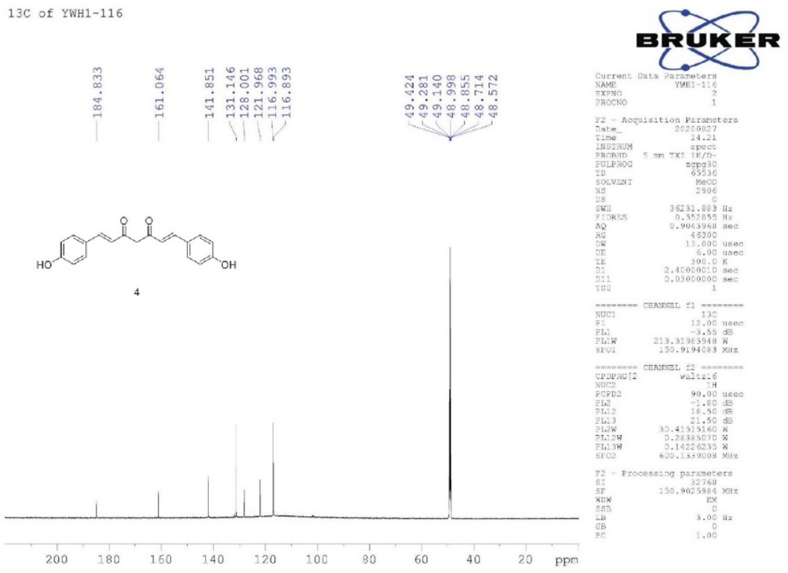

Figure S4. ^{13}C NMR (150 MHz, CD_3OD) of compound **4**.

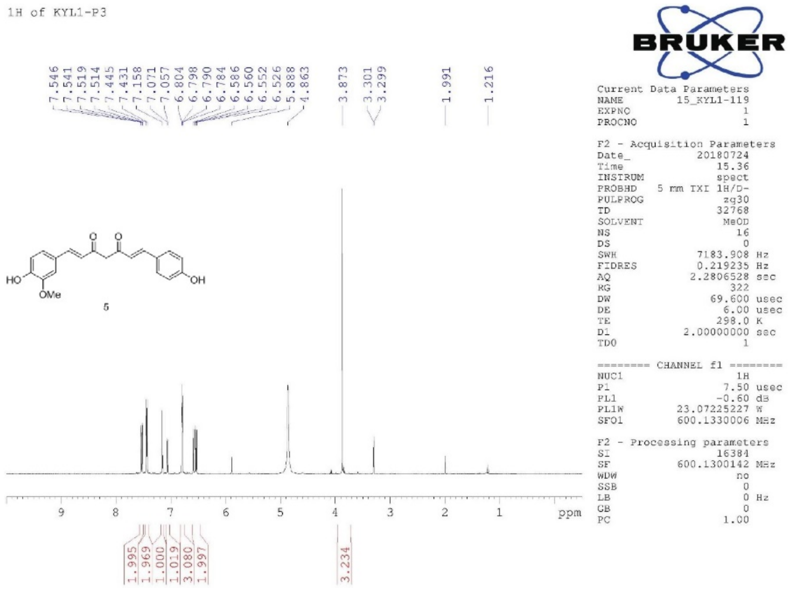

Figure S5. ^{1}H NMR (600 MHz, CD_3OD) of compound **5**.

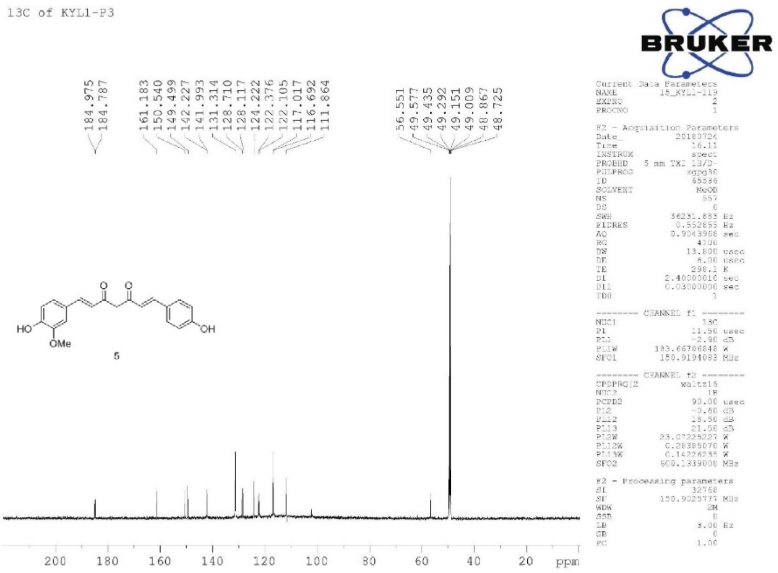

Figure S6. ^{13}C NMR (150 MHz, CD_3OD) of compound **5**.

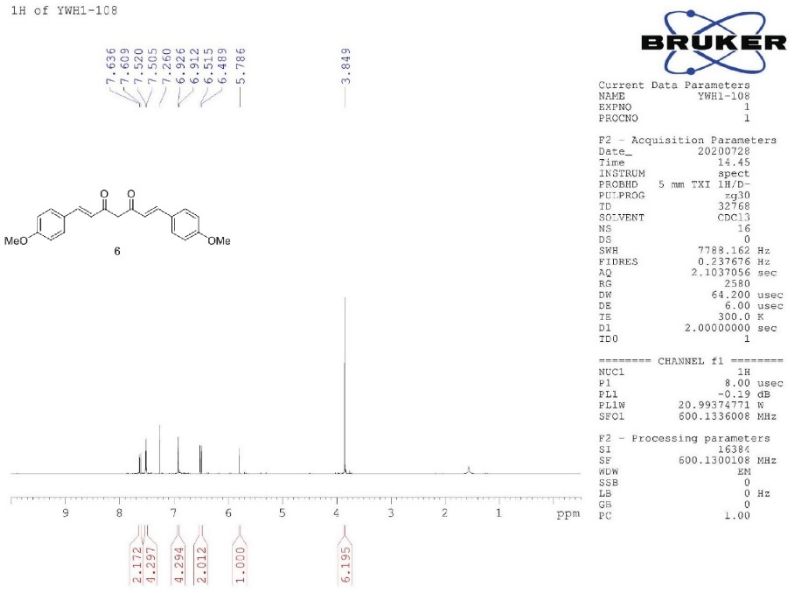

Figure S7. ^{1}H NMR (600 MHz, $CDCl_3$) of compound **6**.

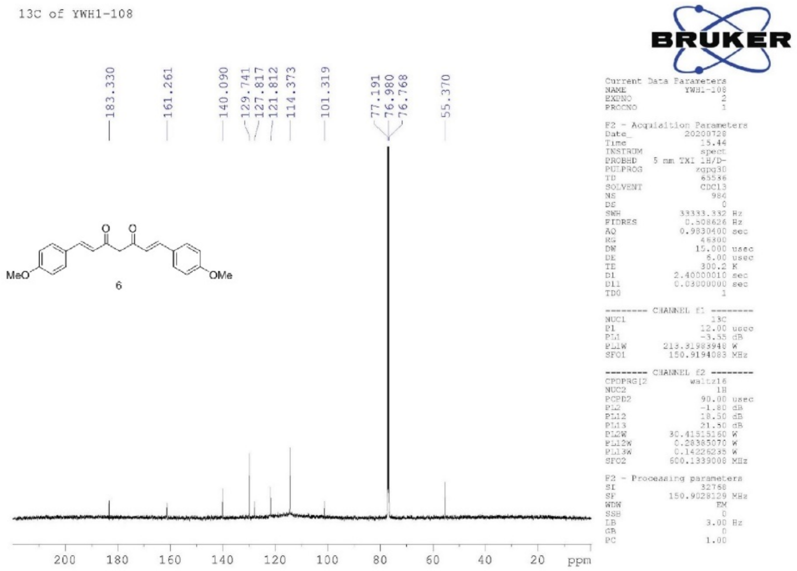

Figure S8. ^{13}C NMR (150 MHz, $CDCl_3$) of compound **6**.

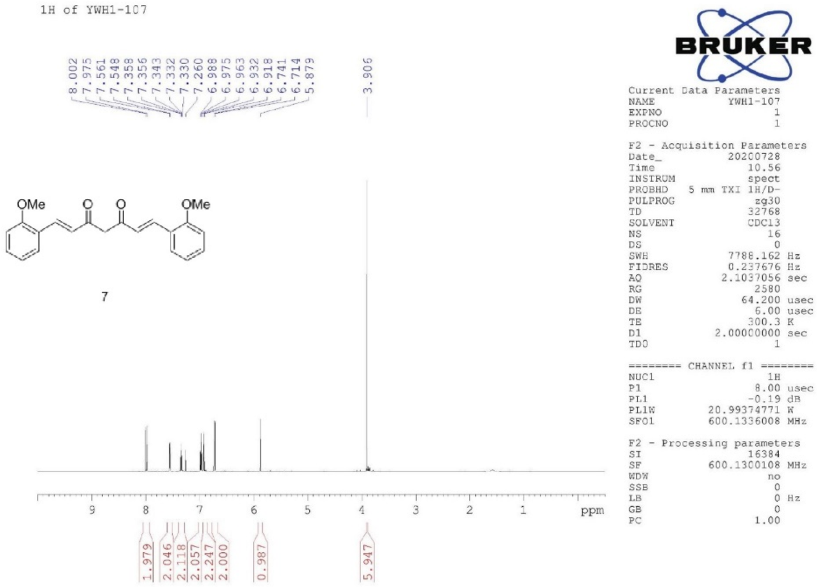

Figure S9. ^{1}H NMR (600 MHz, $CDCl_3$) of compound **7**.

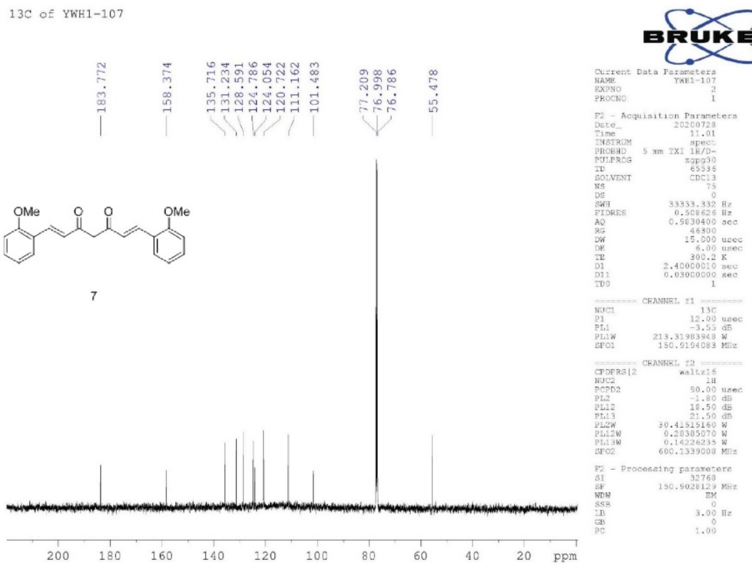

Figure S10. ^{13}C NMR (150 MHz, $CDCl_3$) of compound **7**.

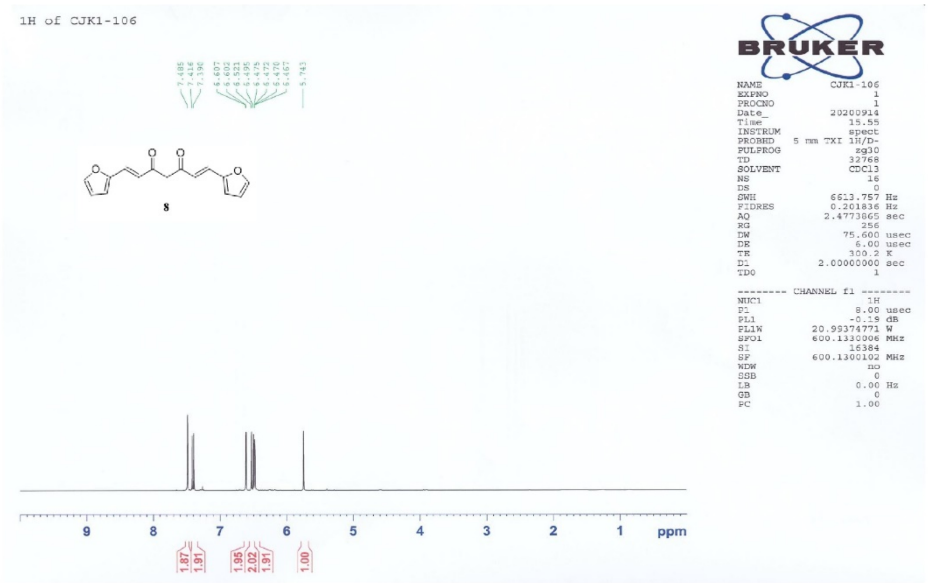

Figure S11. ^{1}H NMR (600 MHz, $CDCl_3$) of compound **8**.

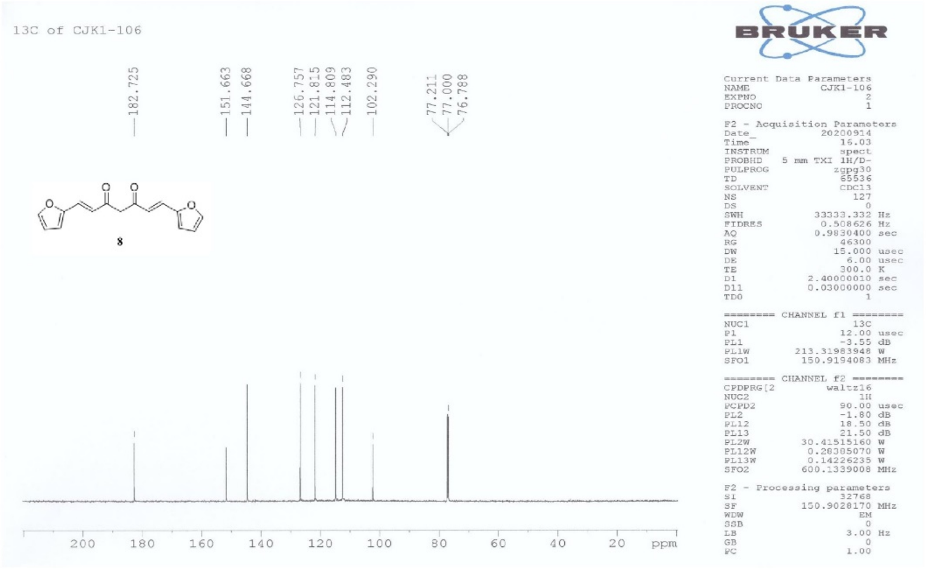

Figure S12. ^{13}C NMR (150 MHz, $CDCl_3$) of compound **8**.

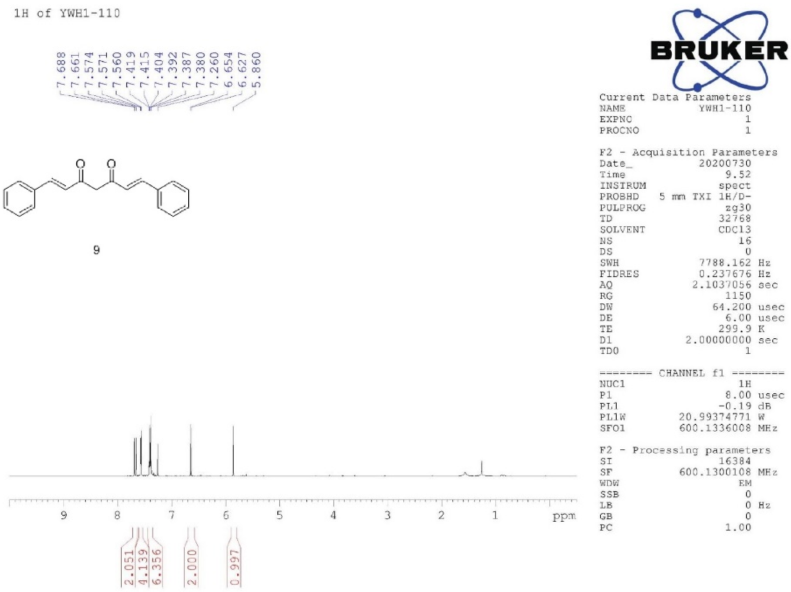

Figure S13. ^{1}H NMR (600 MHz, $CDCl_3$) of compound **9**.

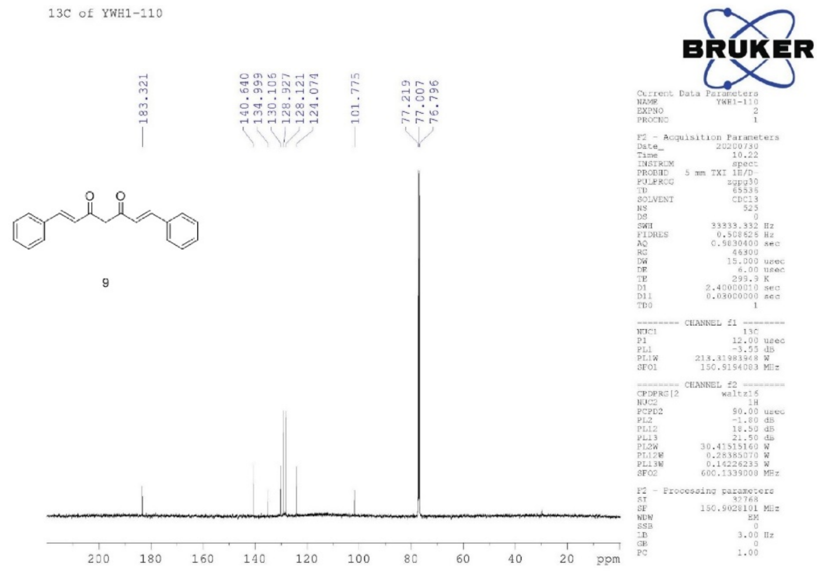

Figure S14. ^{13}C NMR (150 MHz, $CDCl_3$) of compound **9**.

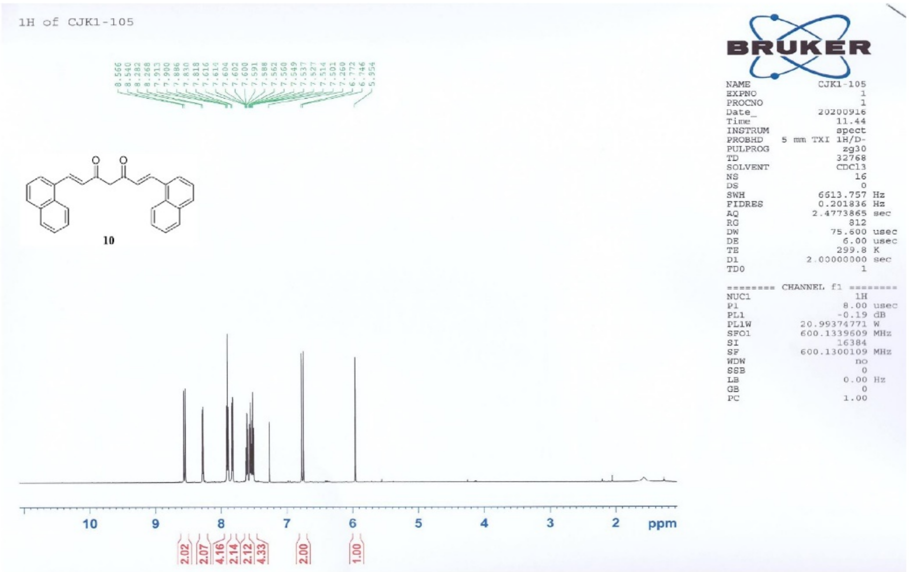

Figure S15. ^{1}H NMR (600 MHz, $CDCl_3$) of compound **10**.

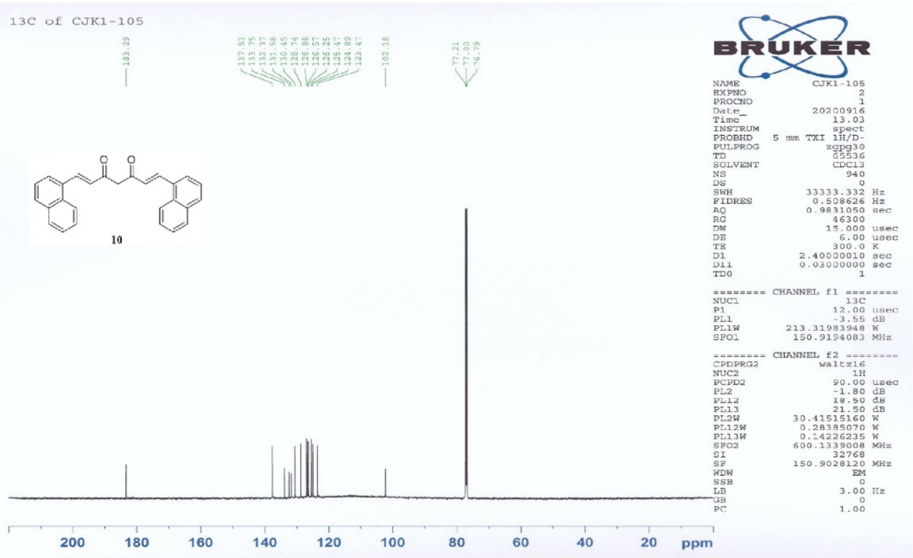

Figure S16. ^{13}C NMR (150 MHz, $CDCl_3$) of compound **10**.

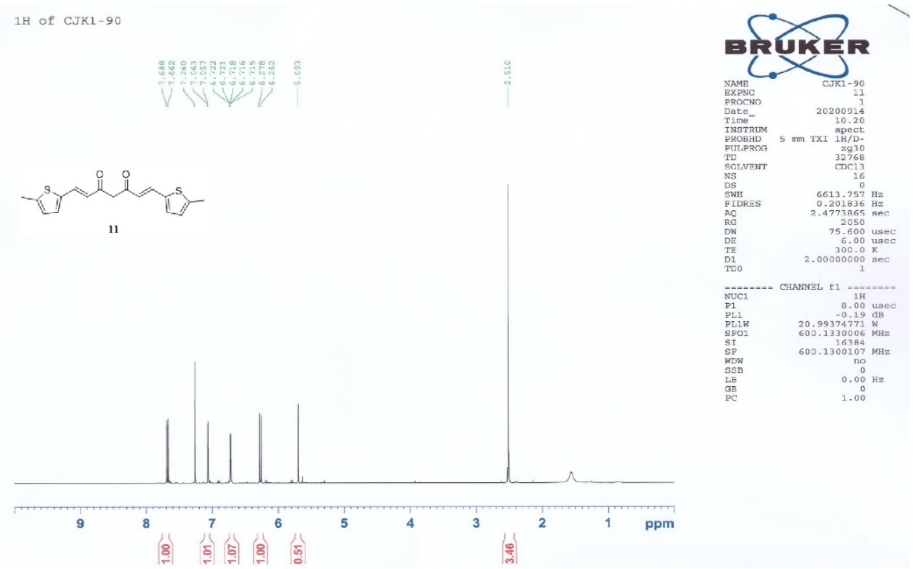

Figure S17. ^{1}H NMR (600 MHz, $CDCl_3$) of compound **11**.

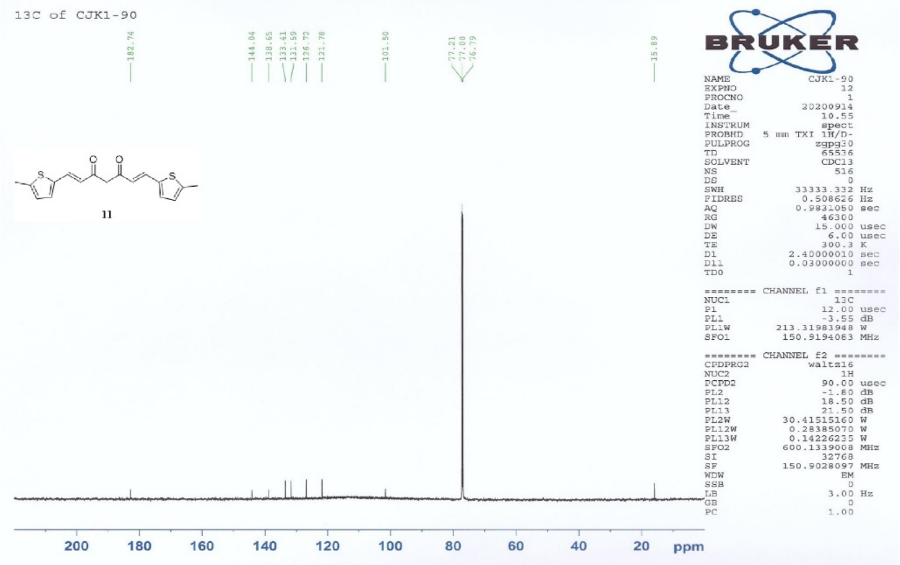

Figure S18. ^{13}C NMR (150 MHz, $CDCl_3$) of compound **11**.

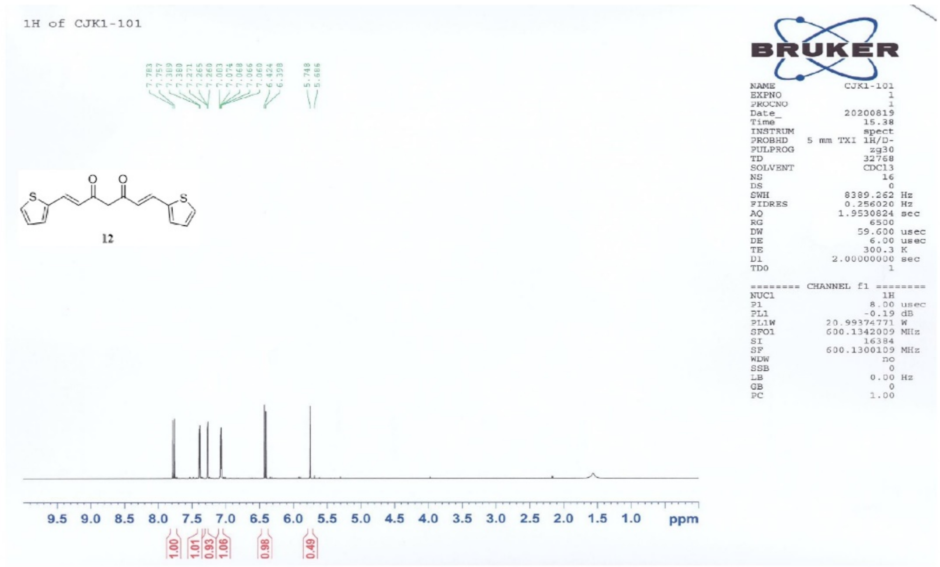

Figure S19. ^{1}H NMR (600 MHz, $CDCl_3$) of compound **12**.

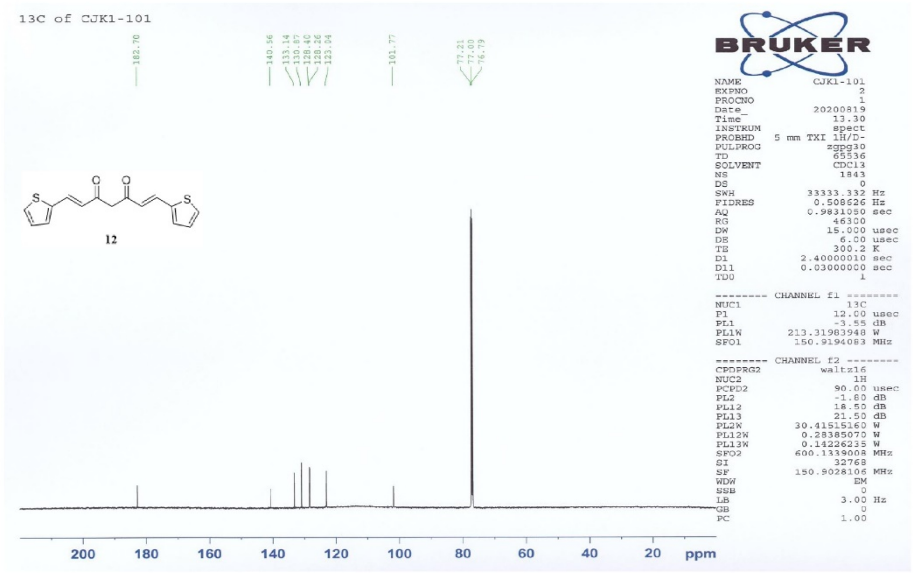

Figure S20. ^{13}C NMR (150 MHz, $CDCl_3$) of compound **12**.

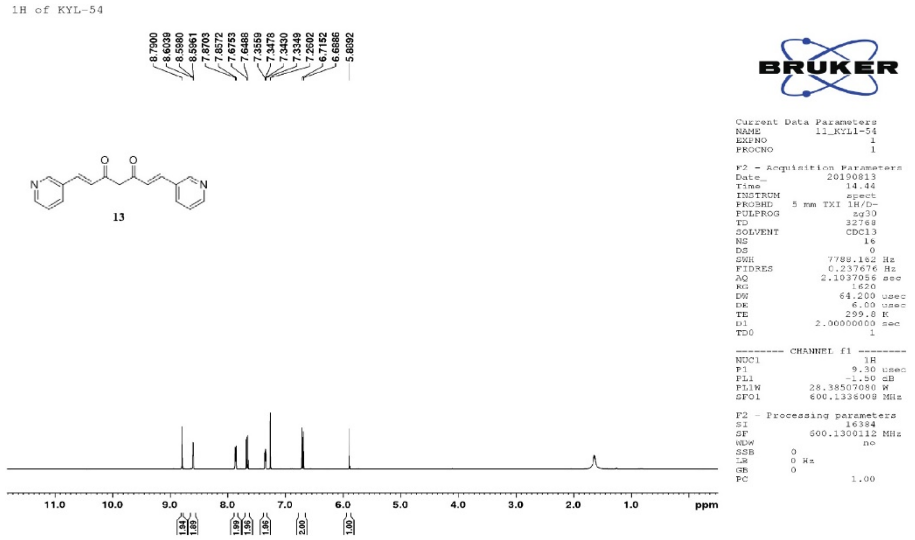

Figure S21. ^{1}H NMR (600 MHz, $CDCl_3$) of compound **13**.

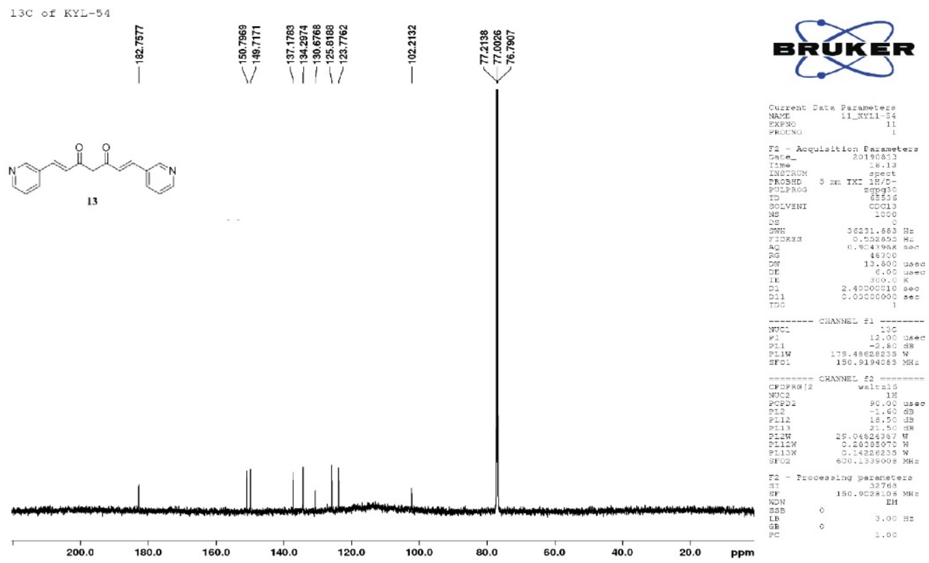

Figure S22. ^{13}C NMR (150 MHz, $CDCl_3$) of compound **13**.

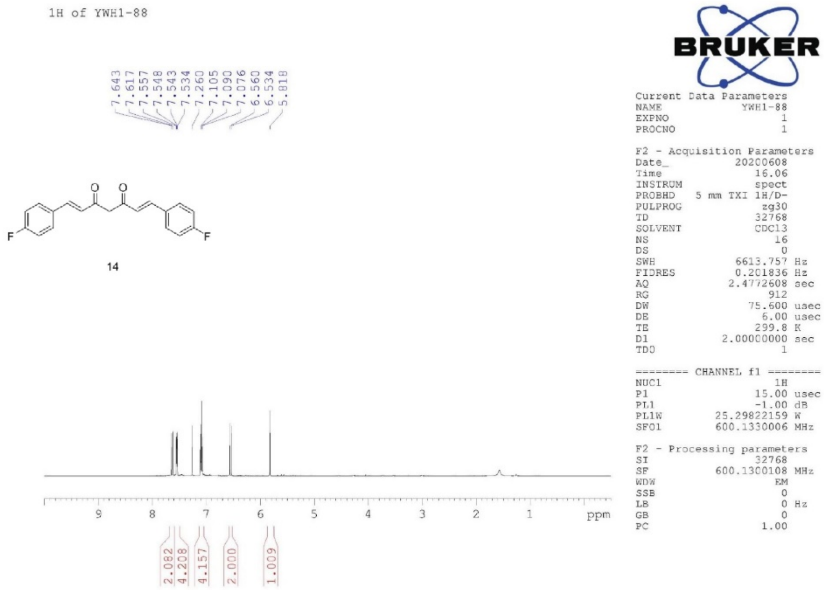

Figure S23. ^{13}C NMR (150 MHz, $CDCl_3$) of compound **14**.

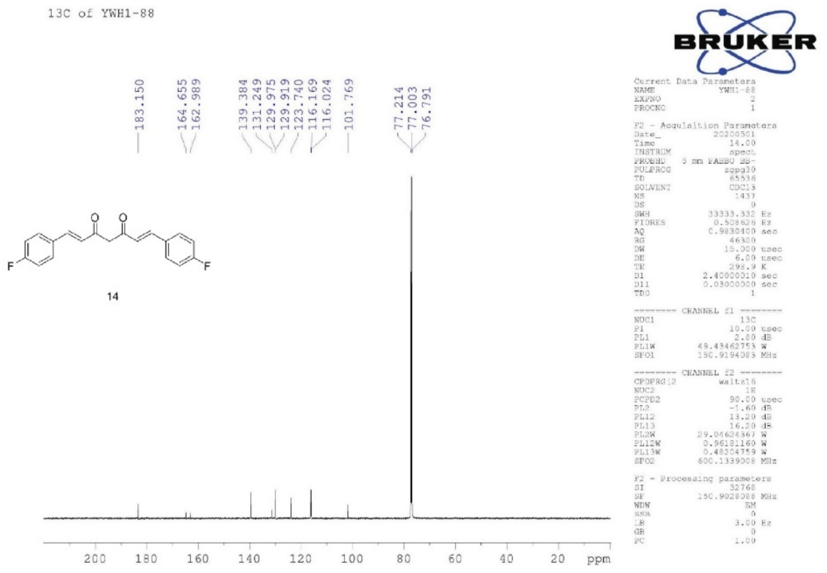

Figure S24. ^{13}C NMR (150 MHz, $CDCl_3$) of compound **14**.

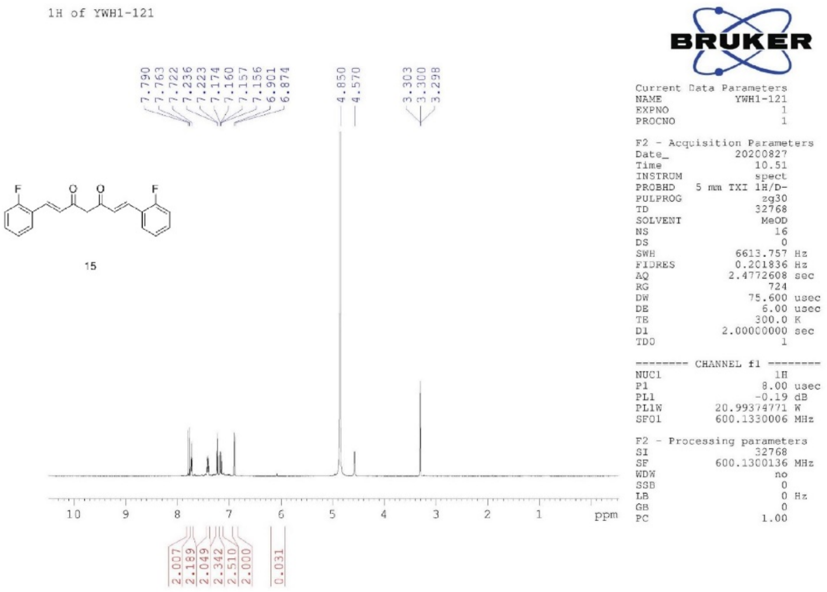

Figure S25. ^{1}H NMR (600 MHz, CD_3OD) of compound **15**.

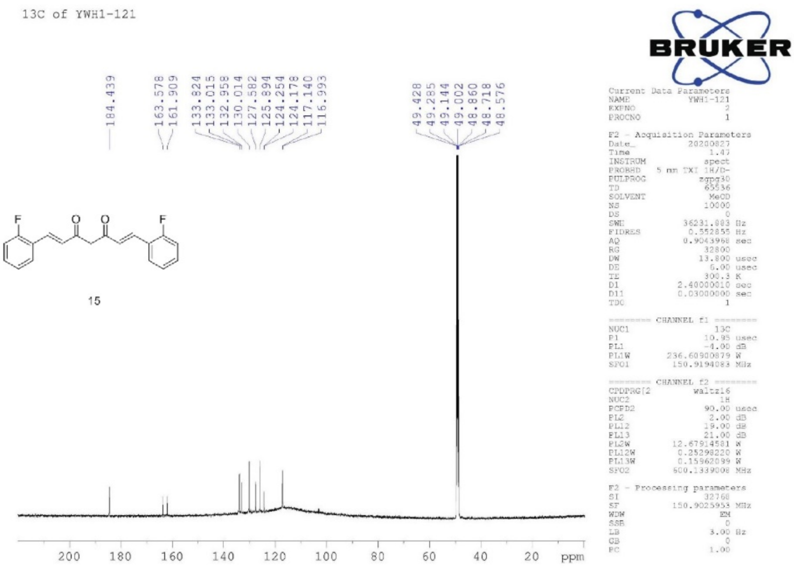

Figure S26. ^{13}C NMR (150 MHz, CD_3OD) of compound **15**.

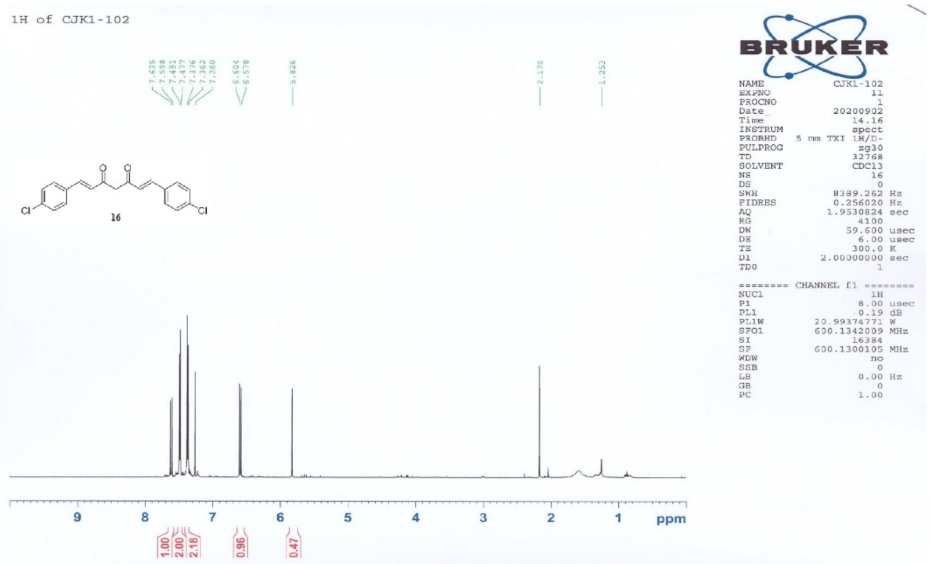

Figure S27. ^{1}H NMR (600 MHz, $CDCl_3$) of compound **16**.

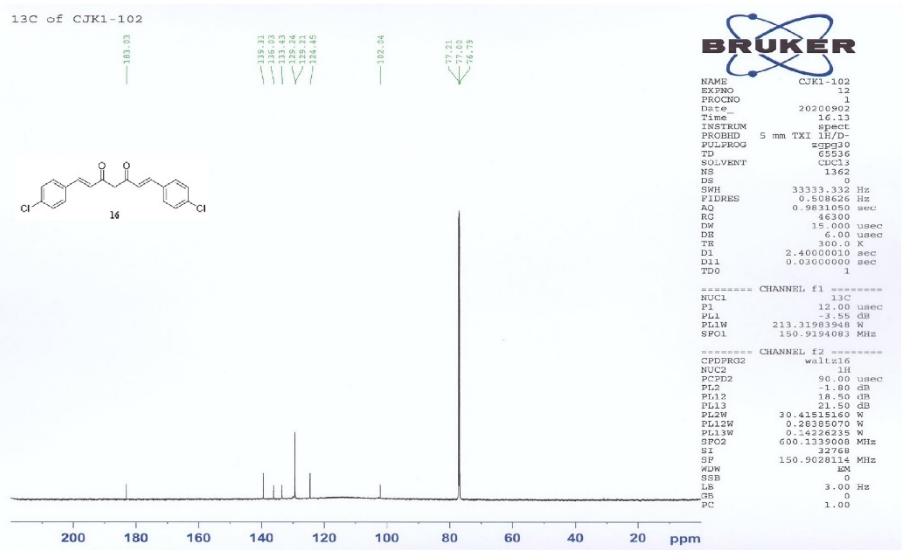

Figure S28. ^{13}C NMR (150 MHz, $CDCl_3$) of compound **16**.

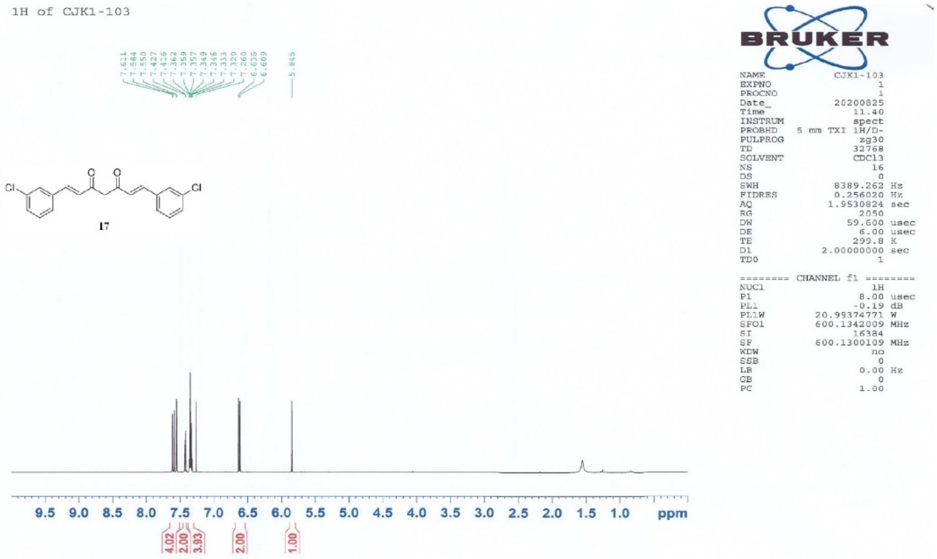

Figure S29. ^{1}H NMR (600 MHz, $CDCl_3$) of compound **17**.

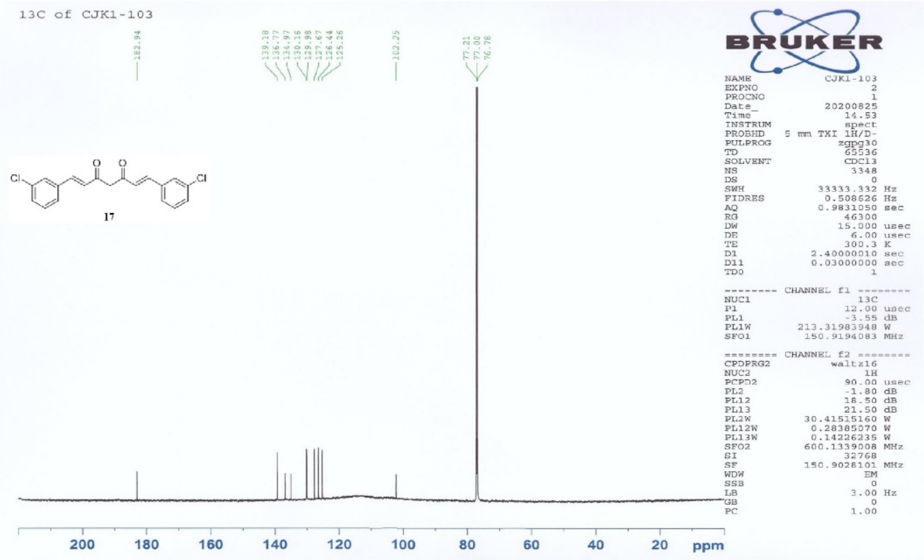

Figure S30. ^{13}C NMR (150 MHz, $CDCl_3$) of compound **17**.

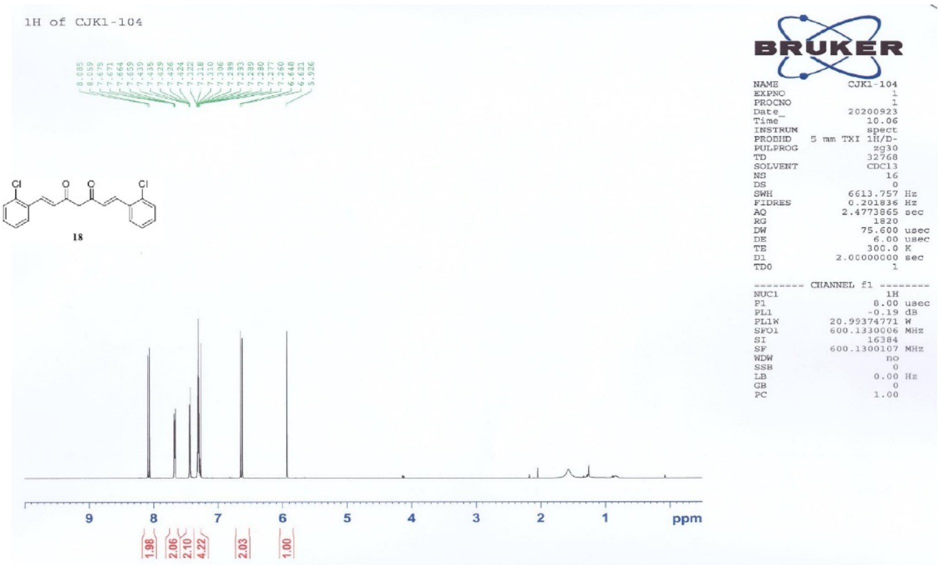

Figure S31. ^{1}H NMR (600 MHz, $CDCl_3$) of compound **18**.

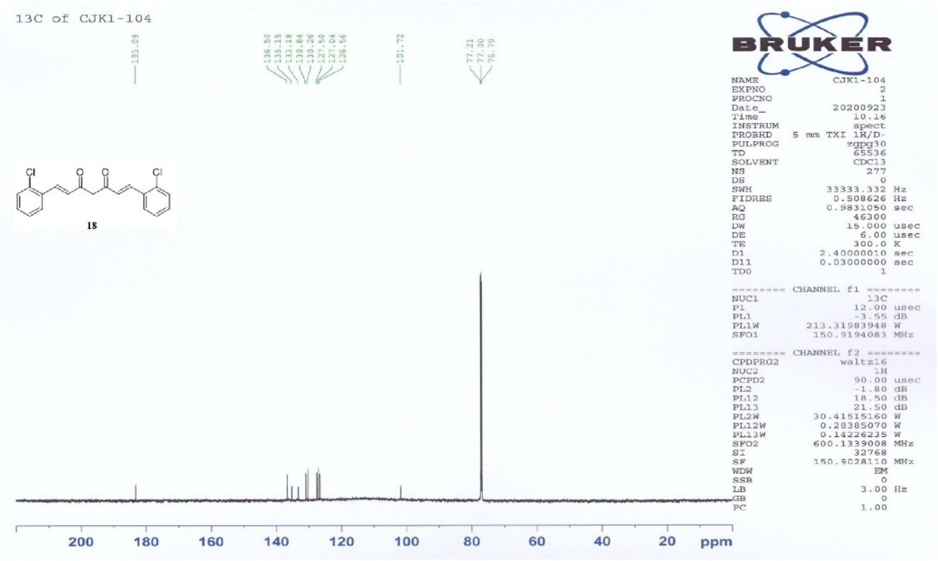

Figure S32. ^{13}C NMR (150 MHz, $CDCl_3$) of compound **18**.

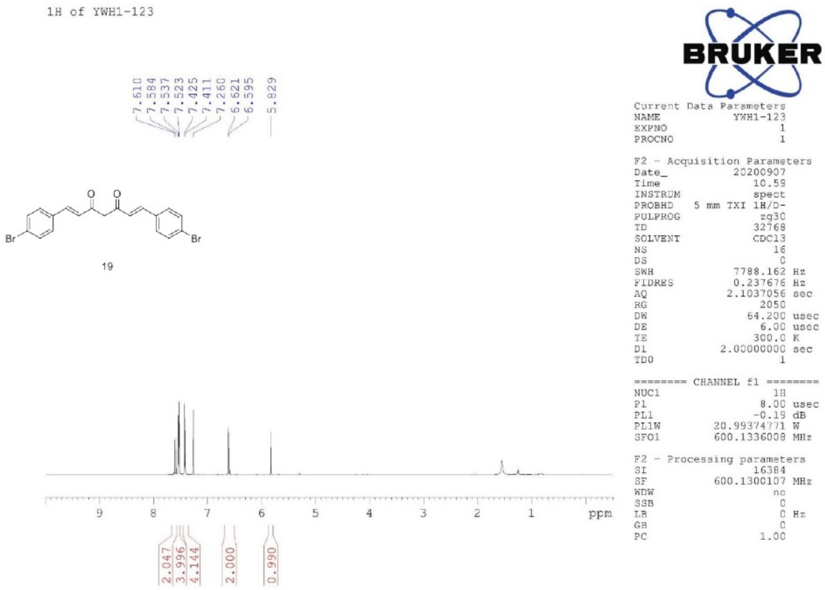

Figure S33. ^{1}H NMR (600 MHz, $CDCl_3$) of compound **19**.

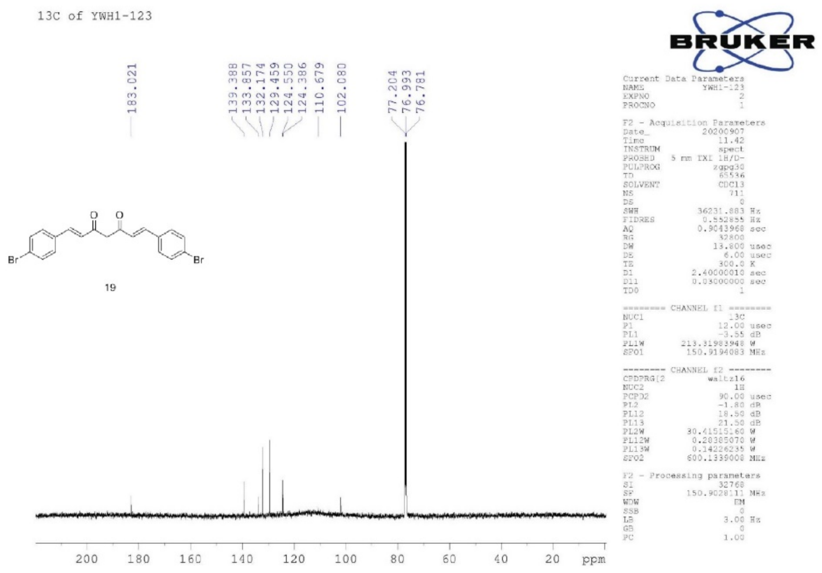

Figure S34. ^{13}C NMR (150 MHz, $CDCl_3$) of compound **19**.

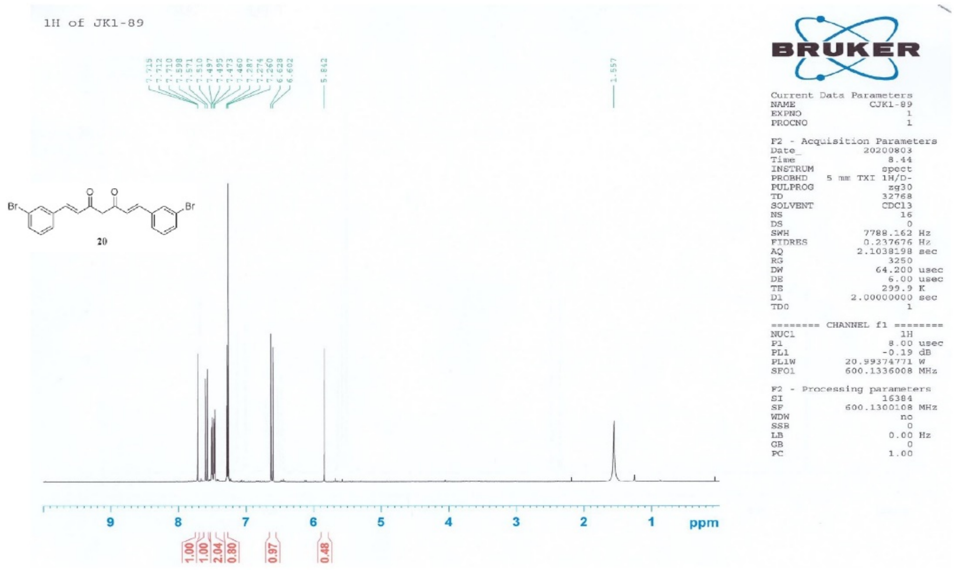

Figure S35. ^{1}H NMR (600 MHz, $CDCl_3$) of compound **20**.

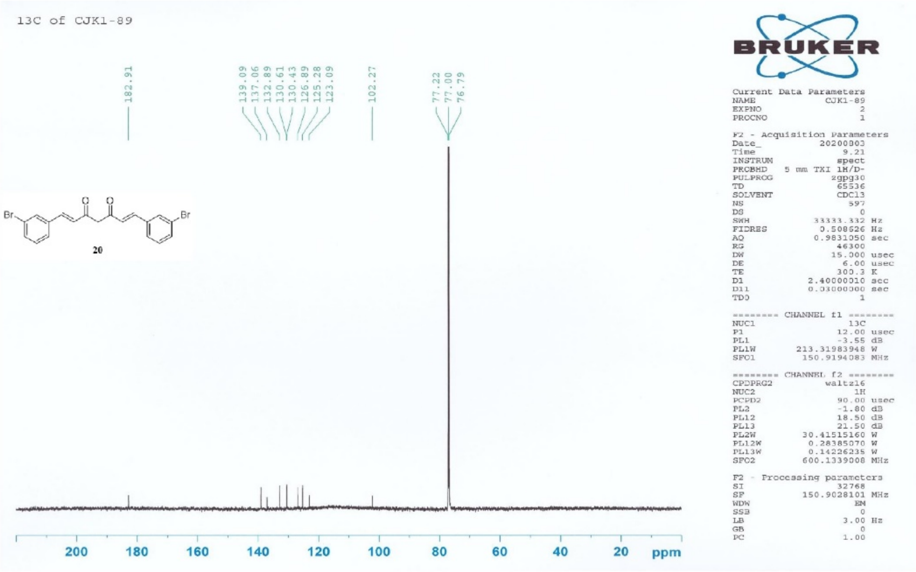

Figure S36. ^{13}C NMR (150 MHz, $CDCl_3$) of compound **20**.

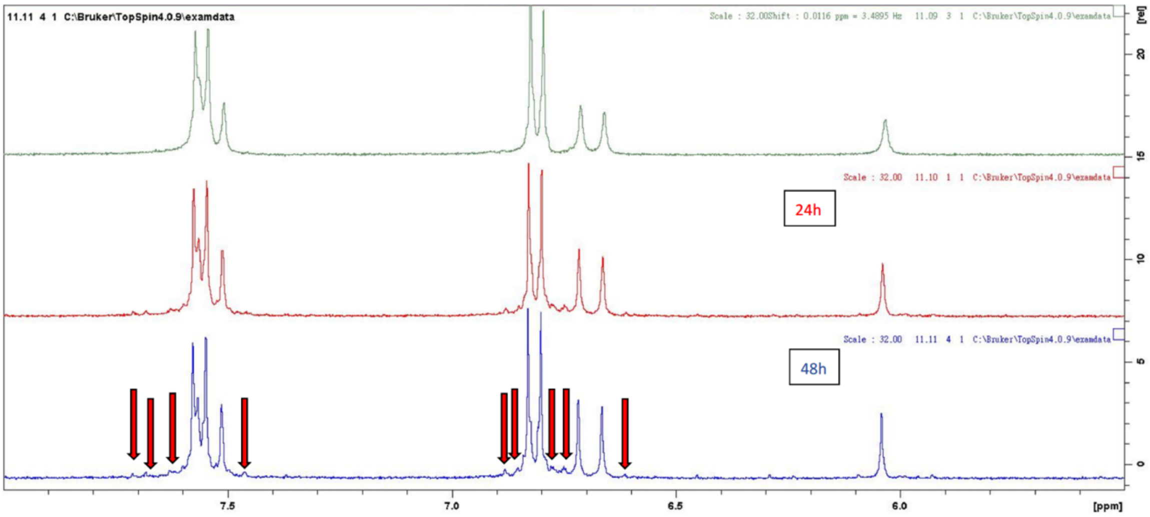

Figure S37. The NMR spectra of degraded compound **4** after storage at room temperature in the dark for 48 h. Arrows indicate the newly formed peaks.
